# Ferroptosis Associates With Diagnosis and Prognosis by Promoting Antitumor Immune Response in Melanoma

**DOI:** 10.3389/fcell.2022.915198

**Published:** 2022-07-08

**Authors:** Benheng Qian, Kui Wu, Xiaoying Lou, kexin Li, Lianpin Wu, Donghong Zhang

**Affiliations:** ^1^ Department of Cardiology, The Second Affiliated Hospital of Wenzhou Medical University, Wenzhou, China; ^2^ State Key Laboratory of Molecular Oncology, Department of Clinical Laboratory, National Cancer Center/National Clinical Research Center for Cancer/Cancer Hospital, Chinese Academy of Medical Sciences and Peking Union Medical College, Beijing, China

**Keywords:** ferroptosis, melanoma, cancer immunity, immunotherapy, pan-cancer

## Abstract

Immunotherapy has greatly improved the clinical benefits of cancer treatment, especially in melanoma. Ferroptosis is a novel mechanism of cell death which relates to immunity. This study aimed at understanding the potential link between ferroptosis and cancer immunocompetent in melanoma using multiple bioinformatics analyses. By the WGCNA assay, we first constructed a key module–gene of ferroptosis, which was strongly correlated with the diagnosis, prognosis, and infiltration of immune cells in melanoma. The elevated module–gene could effectively distinguish melanoma from normal tissues and acted as a good prognostic marker. The module–gene of ferroptosis was positively correlated with the infiltration of immune cells. In particular, the module was positively correlated with the expression of PD-L1 and sensitively increased after effective anti-PD-1 treatment. Furthermore, the differential expression of the module–gene between normal and tumor tissues was observed in pan-cancer. The similarity correlations of the module–gene with infiltration of immune cells and the expressions of PD-L1 were confirmed in the pan-cancer level. Our study demonstrated that the key module–gene of ferroptosis was closely related with diagnosis, prognosis, and anti-immune response in melanoma, as well as in pan-cancer.

## Introduction

With the rapidly emerged research studies on immunotherapy, the immune checkpoint inhibitors like nivolumab and ipilimumab have shown attractive effects in multiple cancers (Topalian et al., 2020; van den Bulk et al., 2018). By binding to immune checkpoints including programmed cell death-1 (PD-1) or cytotoxic T-lymphocyte-associated antigen-4 (CTLA-4), these monoclonal antibodies reactivate the antitumor immune response and bring in profound clinical benefits. To date, the most dramatic effects have been found in metastatic melanoma, which has an increasing incidence and a short historical average survival (Robert et al., 2015). Both KEYNOTE-006 and CheckMate-067 trials showed a median overall survival exceeding 3 years in advanced melanoma using the anti-PD-1 strategy (Hodi et al., 2018; Robert et al., 2019). Moreover, the combination of anti-CTLA4 and anti-PD-1 therapy presents more survival benefits (Larkin et al., 2019). However, the efficacy of these immunotherapies varies widely due to tumor heterogeneity. Thus, understanding the immune status and identifying the biomarkers such as PD-L1, tumor microsatellite instability (MSI), and tumor mutational burden (TMB) is critical for evaluating the efficiency of immunotherapy.

Ferroptosis is an iron-dependent cell death characterized by the oxidative modification of phospholipid membranes (Dolma et al., 2003; Dixon et al., 2012). Morphologically, ferroptosis is marked by shrunk mitochondria with increased bilayer membrane density, ruptured outer membrane, and reduction of crista (Dixon et al., 2012; Conrad et al., 2016; Li et al., 2020). Mechanistically, reactive oxygen species (ROS) launched and ferrous iron (Fe2+) mediated the accumulation of peroxidated phospholipid, leading to the rupture of plasma membrane and cell death (Hassannia et al., 2019; Li et al., 2020). Importantly, the accumulation of iron and ROS production resulting from the high metabolic rate might sensitize cancer to ferroptosis (Dolma et al., 2003; Sosa et al., 2013; Spangler et al., 2016). However, cancer cells may also resist ferroptosis by additional genetic and epigenetic mechanisms, as well as multiple cancer-related pathways, including the most commonly known RAS and TP53 (Chen et al., 2021). Additionally, the previous experimental studies indicated that ferroptosis might interact with cancer immunity. Lipid peroxidation could be derived from the maturation of dendritic cells (DCs) and activation of adaptive immune (Rothe et al., 2015). Interferon-gamma (IFNγ) secreted by CD8^+^ T cells could stimulate the ferroptosis of cancer cells (Wang et al., 2019). Therefore, full understanding of the interaction between ferroptosis and immune activation in tumor tissues may provide a potential strategy for anticancer therapy.

To address how ferroptosis impacts the immunocompetence and clinical outcomes in cancers, we performed multiple bio-informatics analyses and systematically analyzed the role of ferroptosis-related genes in SKCM, which have greatest benefits from immunotherapy and could be a guide to other cancers. We constructed and identified a key module of ferroptosis, which was closely related with the prognosis of SKCM. The elevated expression of the module–gene was positively related with the immune activation and sensitively responds to anti-PD-1 treatment. Notably, the correlation of ferroptosis module and immune activation could be confirmed in the pan-cancer level. Our findings addressed a critical module bridging ferroptosis and cancer immunity, and provide valuable information for immunotherapy.

## Materials and Methods

### Data Collection

A list of total 104 ferroptosis-related genes was obtained from FerrDb (http://www.zhounan.org/ferrdb/) and shown in Supplementary Table S1. The RNA expression datasets of SKCM (472 samples) and other 32 cancer types in TCGA were downloaded from the Xena browser (http://xena.ucsc.edu/) and listed in Supplementary Table S2. Three melanoma datasets were obtained from the GEO database (https://www.ncbi.nlm.nih.gov/geo) for further research, including GSE98394 (Illumina HiSeq 2500, 27 normal samples and 51 tumor samples), GSE65904 (Illumina HumanHT-12 V4.0, 210 samples with clinical data), and GSE91061 (Illumina Genome Analyzer, paired expression data before and after nivolumab treatment from 24 responsers and 18 non-responsers). All the GEO expression data were sorted into the form of log2(FPKM+1) and presented in Supplementary Table S3.

### Weighted Gene Co-Expression Network Analysis

The gene co-expression network analysis was carried out on SKCM samples from TCGA datasets using WGCNA R package according to the official guideline (Langfelder and Horvath, 2008; Langfelder and Horvath, 2012). After a normalized and preliminary sort, the expression matrix was inputted into the WGCNA package. The parameters were set as follows: best soft power threshold, 4; merge cut height, 0.25. Pearson’s correlation analysis was further performed to assess the association between module Eigengenes (ME) and clinical traits as well as immune scores.

### Correlation Analysis Between Module and Gene

The correlation of the expression level between 18 modules and genes was calculated using the Spearman correlation analysis, and the Corrplot package (https://github.com/taiyun/corrplot) was used to visualize the results. A protein interaction between the module and gene was further evaluated by GENEMANIA (http://genemania.org/).

### Hierarchical Clustering Analysis

A total of 16 types of cancer were classified into “hot cancer” and “cold cancer” using the hierarchical clustering analysis based on the expression of PD-L1 and the normalized proportion of lymphocytes infiltrating in the cancer tissues (Teng et al., 2015; Chen et al., 2017). Of note, three cancers (LAML, DLBC, and THYM) were excluded due to their high proportion of immune cells.

### Single Sample Gene Set Enrichment Analysis

To identify the enrichment score of 18 ferroptosis-related module–gene in each sample, we performed the single sample gene set enrichment analysis (ssGSEA) using the GSVA R package (Li et al., 2019; Ye et al., 2019). The Wilcox text was further carried out to evaluate the different levels of enrichment score between tumor and normal groups in the TCGA and GSE98394 databases (Hänzelmann et al., 2013).

### Estimation of Immune Cell Components

The CIBERSORT algorithm is used to quantify the cell fractions in complex tissues based on the gene expression profiles (Newman et al., 2015). In this study, 22 sorted immune cell subtypes were used as a reference set to characterize the relative fractions of immune infiltrating cells in SKCM and other 32 cancer types. After a normalized and preliminary sort, the expression matrix was input to the CIBERSORT algorithm (R package), and 100 permutations were performed. The correlation between the enrichment score of the module–gene and immune infiltrating cells was evaluated using the Spearman analysis.

### Estimation of the Proportion of Tumor-Infiltrating Cells

To explore the correlation between tumor purity and ferroptosis, we applied ESTIMATE (version1.0.13) to calculate the stromal scores and immune scores in each sample (https://R-Forge.R-project.org/projects/estimate/) (Yoshihara et al., 2013). The coefficient between the estimate scores and enrichment score of the module–gene was further performed using the Spearman correlation analysis.

### Microsatellite Instability and Tumor Mutational Burden Estimation in Pan-Cancer

TMB was defined as the total number of somatic nonsynonymous mutations in every one million bases (Chalmers et al., 2017). MSI was determined based on the number of abnormal insertion or deletion events in tumors (Liu et al., 2018). The TMB score and MSI score in each sample were calculated using corresponding simple nucleotide variation data obtained from the TCGA database. The coefficient of the enrichment score with TMB, MSI, and PD-L1 in pan-cancer was calculated by the Spearman correlation analysis and illustrated using radar maps.

### Cox Regression and Survival Analysis

The complete clinical information, including pathological and prognostic data, was obtained from corresponding datasets. Univariate and multivariate Cox regression were then performed using the “coxph” function of the “survival” package. For survival curves, samples were divided into two groups according to the levels of the enrichment score; the Kaplan–Meier estimate was then performed using the “survival” and “survminer” packages. To establish the risk model, the least absolute shrinkage and selection operator (LASSO) regression was performed using the “glmnet” package.

### Statistical Analysis

Statistical analyses were performed using R software (version 3.6.2). Continuous variables were expressed as the mean ± standard error and analyzed using Student’s t tests or Wilcoxon tests. The Chi-square test and Fisher’s exact test were used to analyze categorical variables. The coefficient of the module–gene with TMB, MSI, and PD-L1 was evaluated using the Spearman correlation analysis. A two-side *p* value of <0.05 was deemed to be statistically significant in this study.

## Results

### Construction and Identification of the Key Module of Ferroptosis in SKCM

**FIGURE 1:**
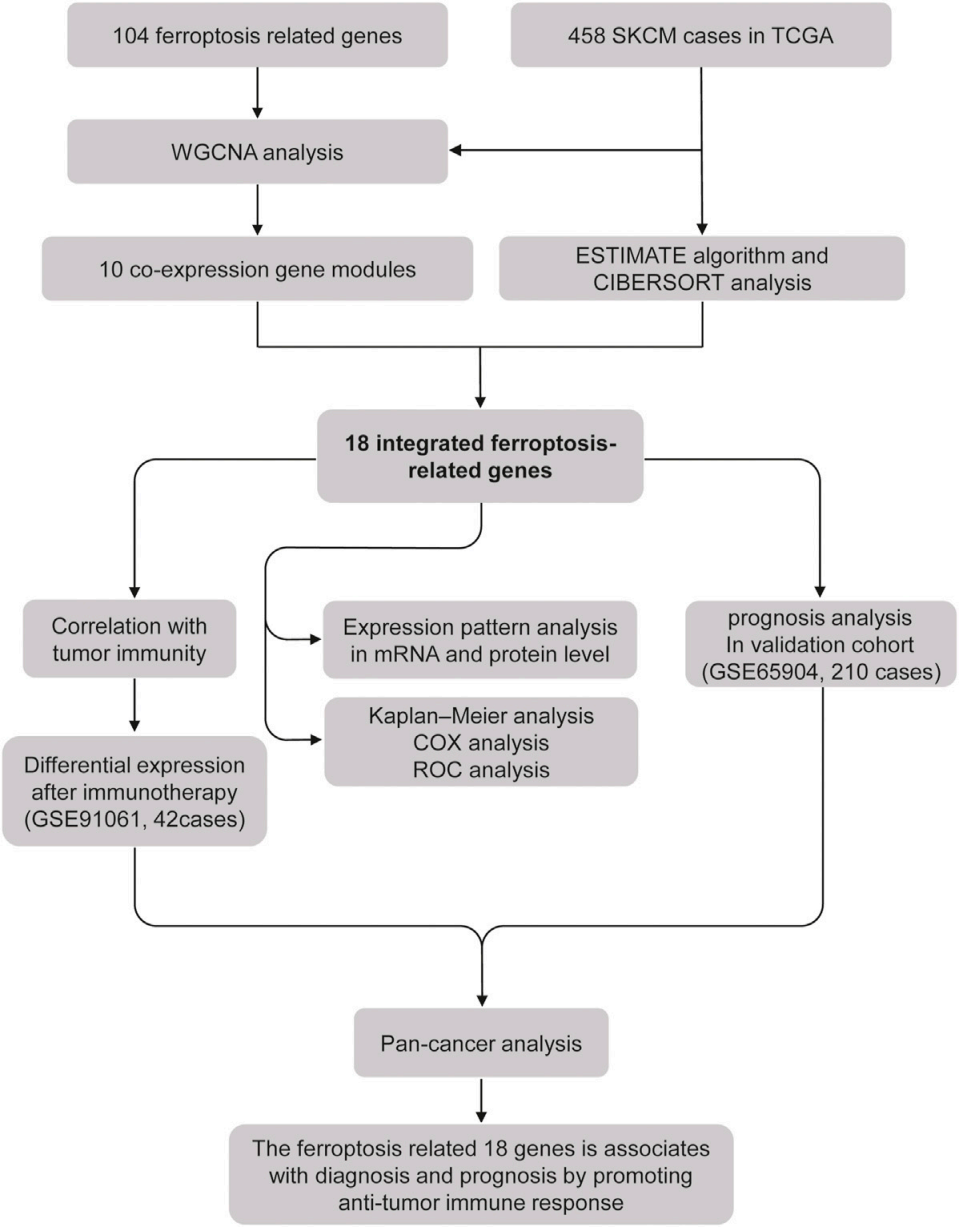
Flowchart of the study.

**FIGURE 2:**
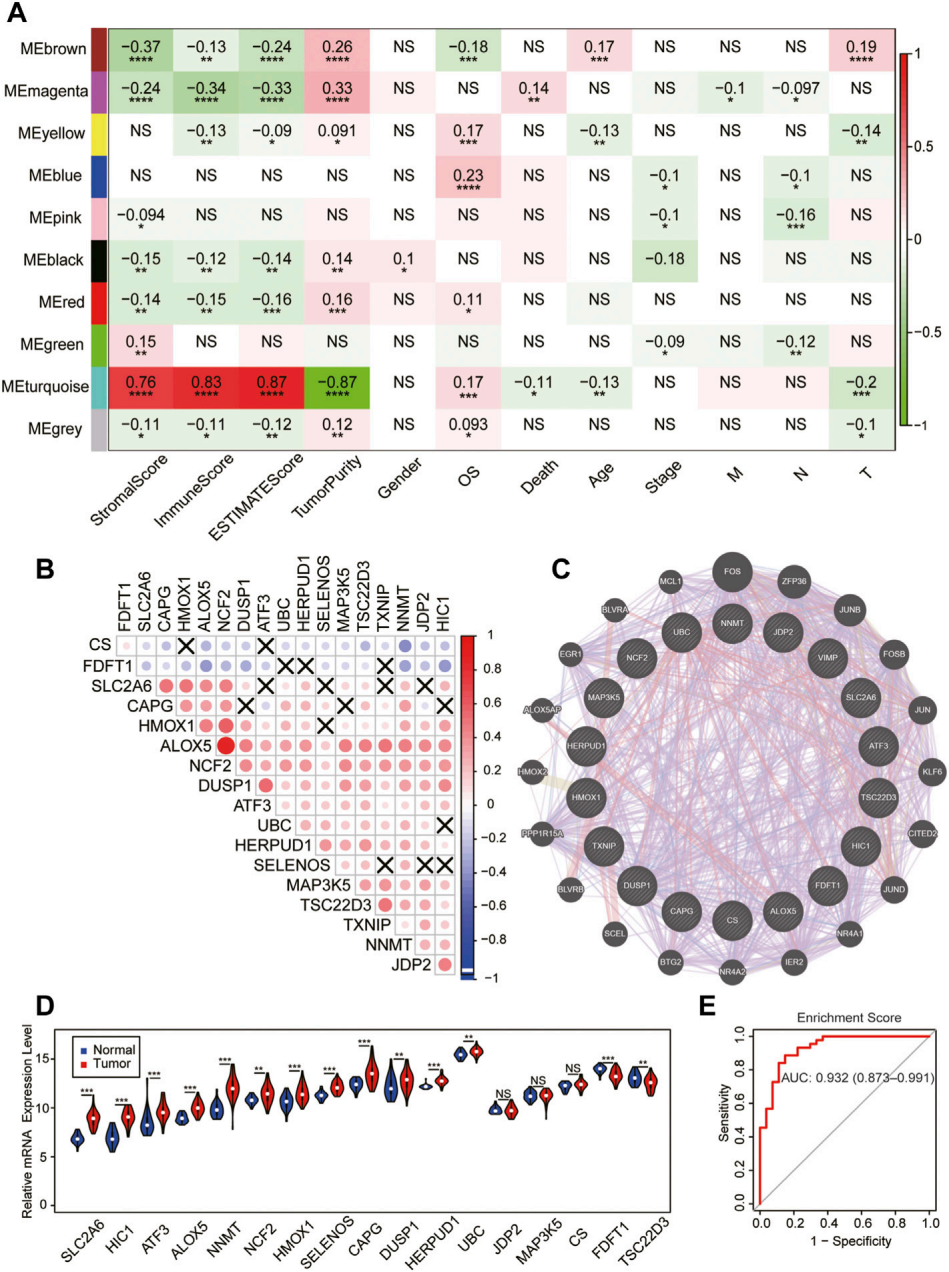
Identification of the key module of ferroptosis in SKCM. **(A)** The gene–module trait relationship based on the weighted gene correlation network analysis (WGCNA) in SKCM. **(B)** The mRNA expression correlation heatmap of the turquoise module–gene in the TCGA database. X, no significant correlations. **(C)** Weighted interaction networks of genes in the turquoise module performed by GENEMANIA. **(D)** Differential expression of 17 module–gene between normal and tumor tissue in the GSE98394 database. The violin filling in red represents tumor samples, while blue represents normal tissues. **(E)** The ROC curve of the enrichment score in the GSE98394 database. *p < 0.05; **p < 0.01; ***p < 0.001; and ****p < 0.0001 was determined by the Spearman analysis and the Wilcoxon rank-sum test in **(D)**

The current study’s flow diagram is displayed in Figure 1. To hunt the key gene module of ferroptosis involved in SKCM, we conducted a co-expression network of 105 ferroptosis-related genes in 458 cases of SKCM samples obtained from the TCGA database (Supplementary Figures S1, S2). A total of 10 colored modules were identified, and their correlation with clinical traits is shown in Figure 2A. Interestingly, we found the turquoise module was strongly correlated with the overall survival (OS, coefficient = 0.17, *p* < 0.001), death (coefficient = −0.11, *p* < 0.05), and tumor size (coefficient = −0.2, *p* < 0.001). Of note, the turquoise module was also positively correlated to the stromal score (coefficient = 0.76, *p* < 0.0001), immune score (coefficient = 0.83, *p* < 0.0001), estimate score (coefficient = 0.87, *p* < 0.0001), but negatively related to tumor purity (coefficient = −0.87, *p* < 0.0001). Consistent with the co-expression pattern of genes in the turquoise module, strong interactions were observed between individual genes at mRNA and protein levels using the GENEMANIA assay (Figures 2B,C). Moreover, most genes in the turquoise module indicated the activation of ferroptosis according to the previous reports (Supplementary Table S1), indicating ferroptosis is associated with the prognosis of SKCM.

Next, we tested whether the expression of the module–gene variated during the development of SKCM in GSE98394. Interestingly, we found the enrichment score of the module–gene and expression of most individual genes were increased in cancer tissues compared with controls (Figure 2D, Supplementary Figure S1C). In addition, the up-regulated enrichment score had a better diagnostic value for SKCM patients (AUC = 0.932, Figure 2E). Thus, we addressed the turquoise module as a key ferroptosis-related gene cluster, which was closely related with cancer immunity, diagnosis, and prognosis of SKCM.

### Confirmation of the Prognostic Value of the Module–Gene in SKCM

**FIGURE 3:**
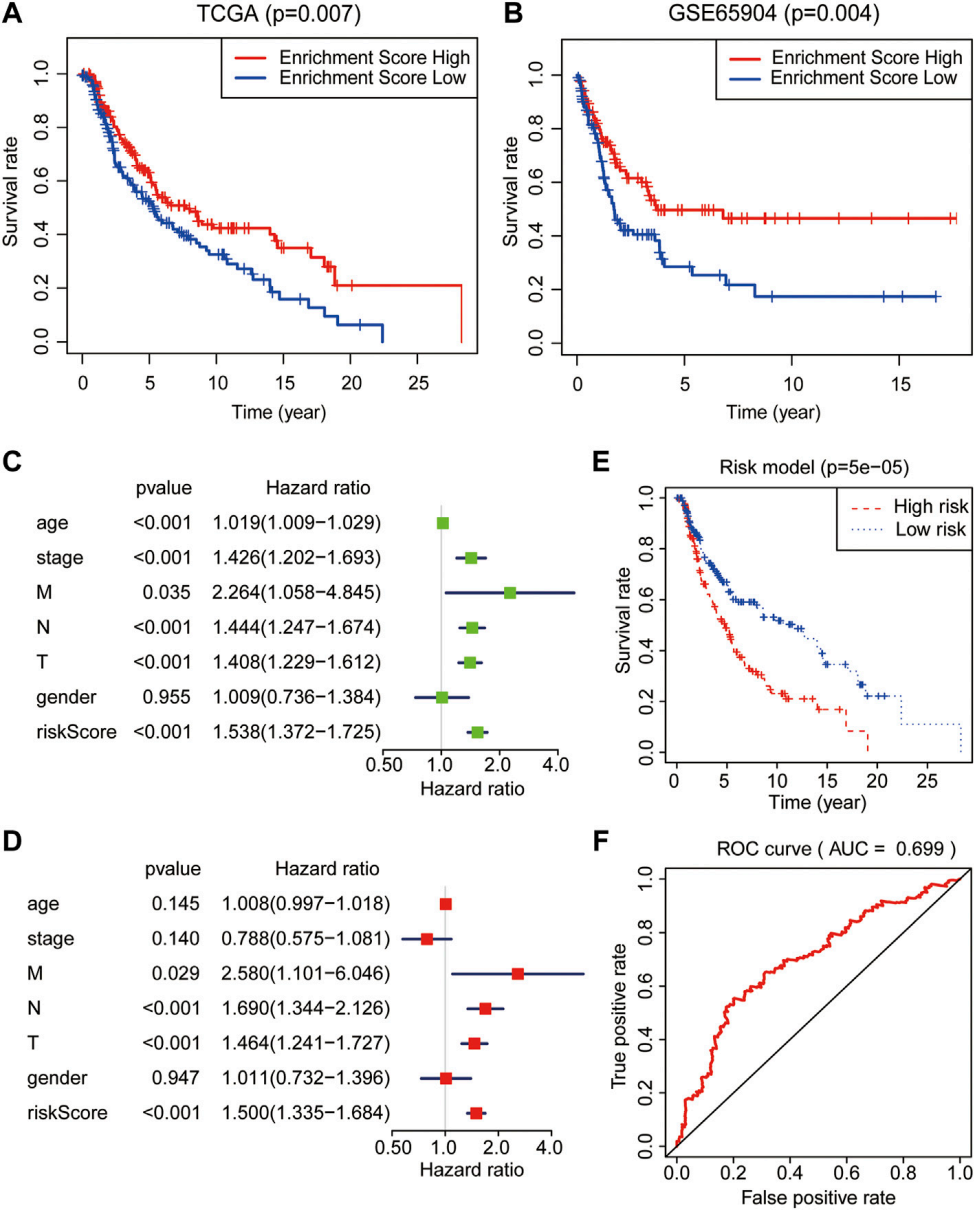
Prognostic analysis of the module–gene of ferroptosis in SKCM. The Kaplan–Meier survival curves in the TCGA **(A)** and GSE65904 databases **(B)**. The enrichment score act as a protect factor in both the training cohort and validation cohorts. Univariate **(C)** and multivariate **(D)** Cox regression analyses of the risk model obtained from the LASSO‐logistic regression analysis in the TCGA database. *p* values were calculated using Fisher’s exact test. **(E)** The Kaplan–Meier survival curve showed the survival benefits in the low-risk group. **(F)** The receiver operating characteristic curve (ROC) curves for the prediction of 5-year survival rates using the risk model in SKCM.

To further testify the prognostic value of the turquoise module, SKCM samples with clinical information were obtained from the TCGA and GSE65904 database as the training and validation cohort, respectively. Interestingly, as displayed in Figures 3A,B, a high level of the module–gene enrichment score indicated the worse outcome of SKCM in both cohorts. We next performed the LASSO‐logistic regression analysis to establish a risk model for of SKCM based on the expression of the module–gene (Supplementary Figures S2A,B). The risk score showed a powerful and independent survival predictor of SKCM after the adjustment for clinicopathological variables, including age, gender, TNM, and cancer stage. In addition, the TNM stage worked as the co-factor with the risk score and predicated the worse prognosis (Figures 3C,D). The K-M survival curve showed significant survival benefit in the low-risk group (*p* = 0.00005, Figure 3E). Consistently, the ROC curve also demonstrated that the risk model performed well in predicting the 5-year survival rates of SKCM (AUC = 0.699, Figure 3F). Similar to the enrichment score of the module–gene, the individual genes, such as SELENOS, JDP2, UBC, ALOX5, HERPUD1, NCF2, MAP3K5, TSC22D3, and NNMT, also showed protective factors in SKCM (Supplementary Figure S2C). The Kaplan–Meier survival curve further proved that ALOX5, NCF2, SELENOS, and HERPUD1 worked as protecting factors in both TCGA and GSE65904 databases (*p* < 0.05, Supplementary Figures S2D–K).

### The Turquoise Module Correlates With Immune Activation in SKCM

**FIGURE 4:**
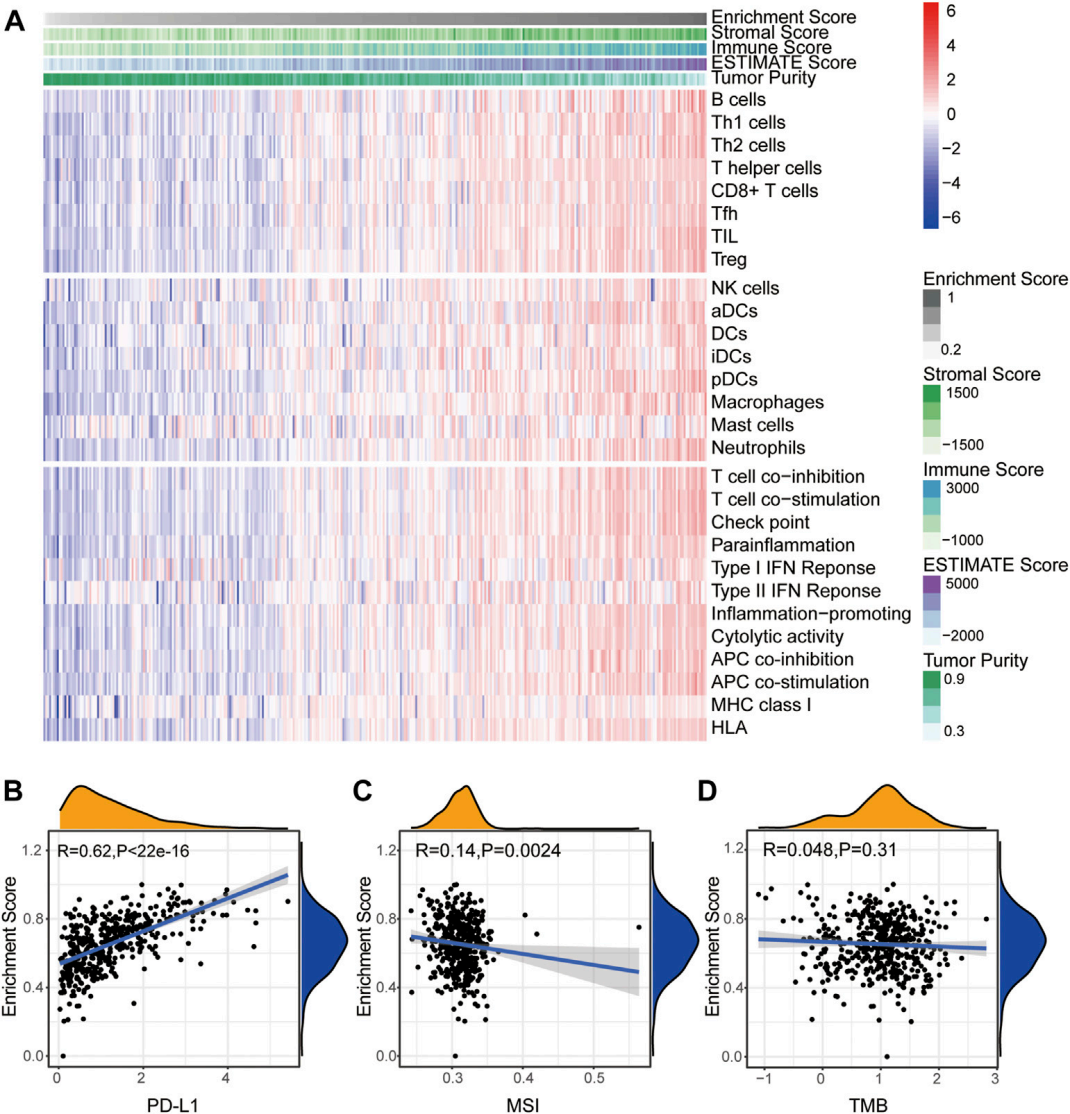
Turquoise module correlates with immune activation in SKCM. **(A)** Heatmap of the enrichment score and infiltration of 28 types of immune cells as well as immune scores. **(B**–**D)** Correlation between the enrichment score of the module–gene and PD-L1 mRNA expression **(B)**, MSI **(C)**, and TMB **(D)**. The coefficient was calculated by the Spearman analysis.

We next calculated the infiltration of immune cells in each SKCM sample and clustered the samples base on the enrichment score of the module–gene. As shown in Figure 4A, the enrichment score was positively correlated with the stromal score, immune score, ESTIMATE score, and individual immune cell numbers, but negatively correlated with tumor purity, suggesting good correlation between the module–gene and the infiltration of immune cells. Interestingly, we found the turquoise module was positively correlated with the expression of PD-L1 in the mRNA level (R = 0.62, *p* < 0.0001, Figure 4B) and negatively related with MSI (R = −0.14, *p* = 0.0024. Figure 4C), which might attribute to escaping from immunosurveillance and implying the module–gene as indicators of immunotherapy (Langfelder and Horvath, 2008). However, no significant correlation was found between the module–gene and TMB score (Figure 4D). Aforementioned observations suggested that ferroptosis might trigger cancer immune activity independent from genetic mutational burdens.

### The Turquoise Module Responses to the Immunotherapy by anti-PD-1

**FIGURE 5:**
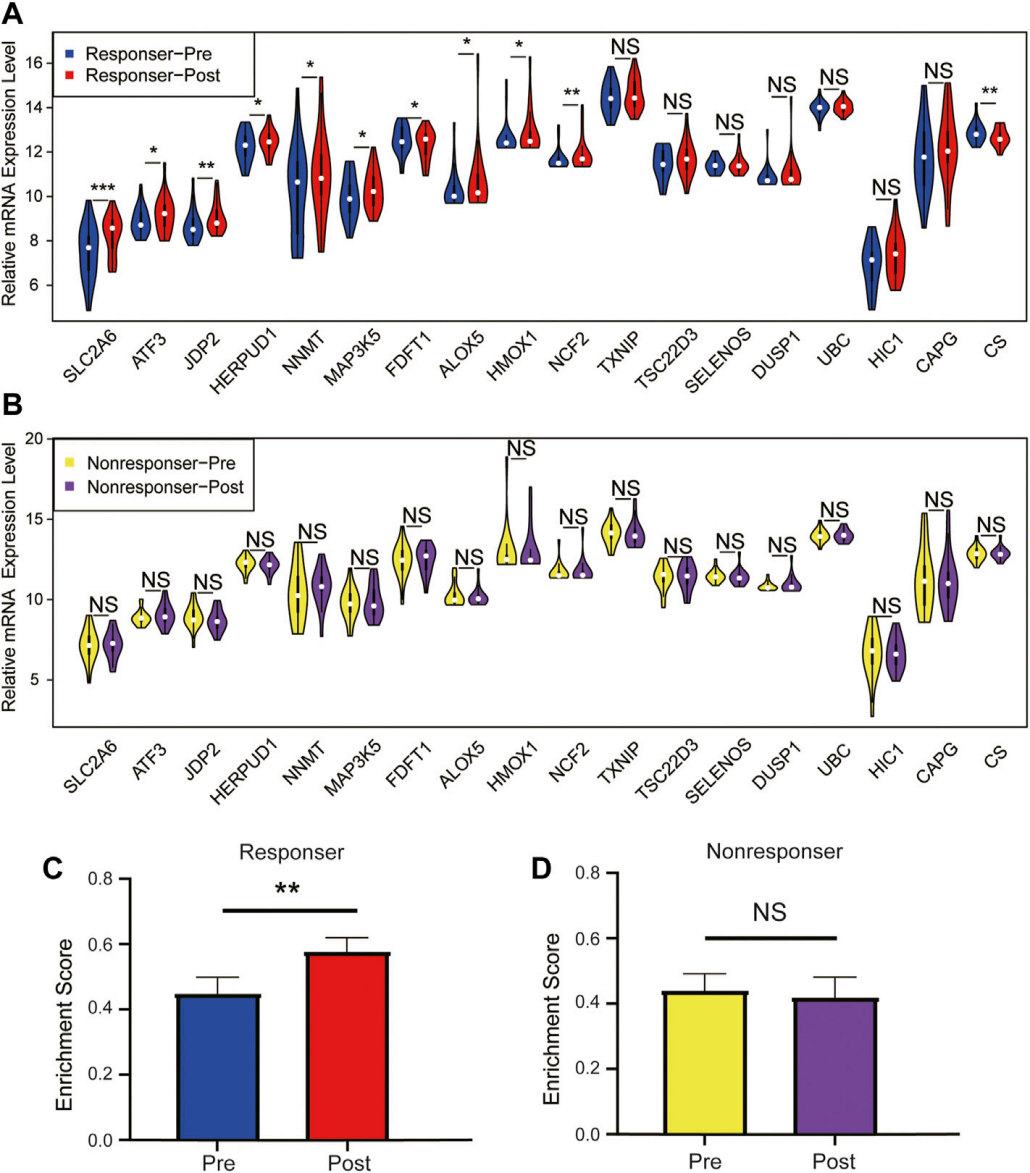
Relative mRNA expression level of module–gene between patient pre- and post-nivolumab treatment. Differential expression of the individual module–gene in patients before and after anti-PD-1 treatment in the response group **(A)** and the nonresponse group **(B)**. The mRNA expression was calculated in the form of log2(FPKM+1) and presented as violin plots. The whole module gene expression (enrichment score) of ferroptosis in patients before and after anti-PD-1 treatment in the response group **(C)** and nonresponse group **(D)**. Data were expressed as the mean ± standard error and analyzed using Student’s t test (*p < 0.05; **p < 0.01; and ***p < 0.001).

To further investigate the module–gene response to immunotherapy, we next evaluated the differential expression of the module–gene after immunotherapy in SKCM. A total of 42 patients (24 responsers and 18 non-responsers) with nivolumab treatment were obtained from GSE91061. As a result, in the response group, the whole turquoise module and more than 60% of genes including SLC2A6, ATF3, JDP2, HERPUD1, NNMT, MAP3K5, FDFT1, ALOX5, HMOX1, and NCF2 were up-regulated after anti-PD-1 therapy (Figures 5A,C). Interestingly, no significant changes of the module–gene were observed in the nonresponse group (Figures 5B,D). These results were highly consistent with those of our previous studies in TCGA and GSE65904 databases, especially ALOX5, HERPUD1, and NCF2. Collectively, we concluded that the ferroptosis-related module–gene might promote the immune activation especially after immunotherapy, and contribute to the good prognosis of SKCM.

### The Correlation of the Module–Gene With Cancer Immunity Is Confirmed in Pan-Cancer

**FIGURE 6:**
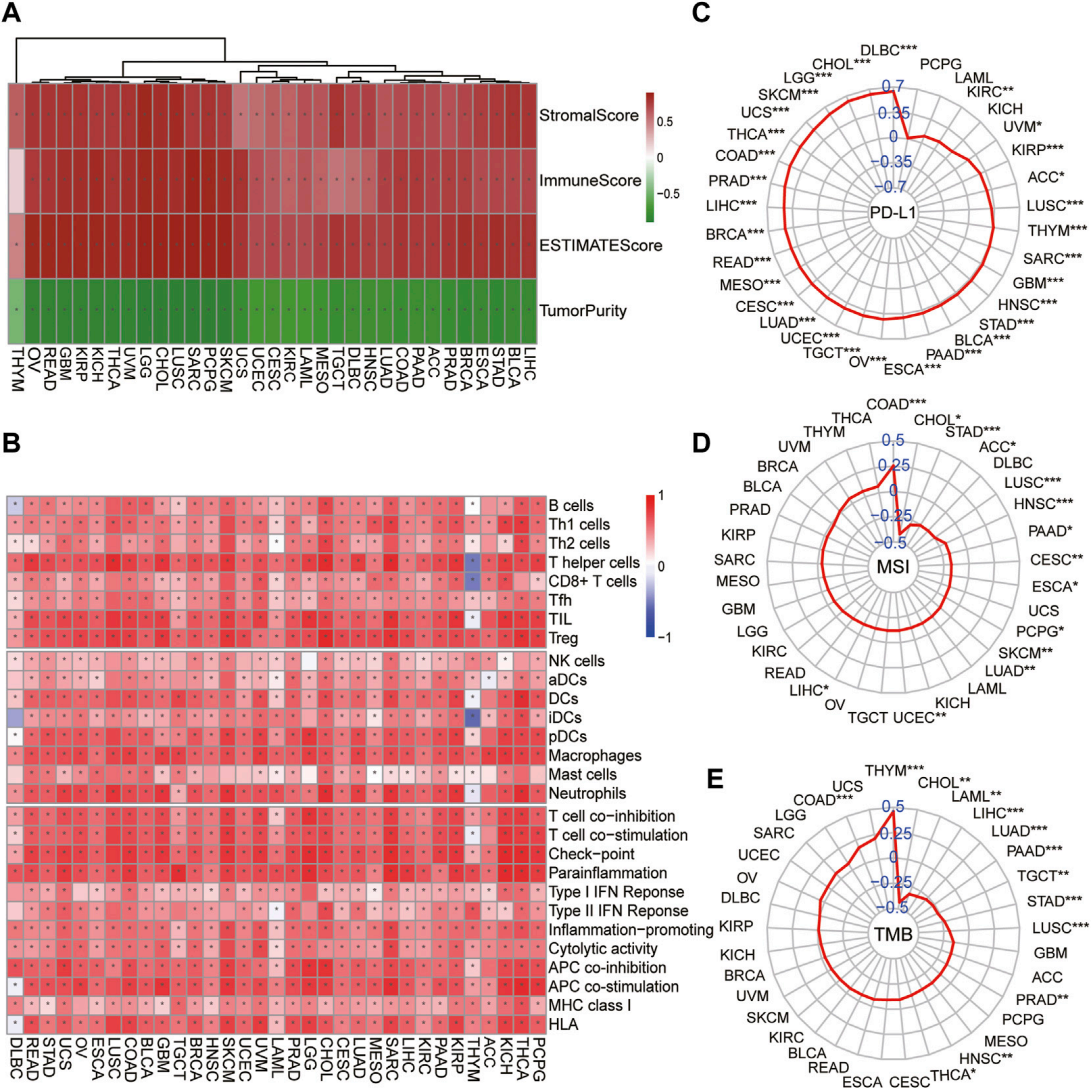
Correlation of ferroptosis-related module–gene with immunity in pan-cancer. Heatmap of the enrichment score and immune scores **(A)** or 28 types of immune cells **(B)** in 33 types of cancer. *p < 0.05 was calculated using the Spearman analysis. The correlation of the enrichment score and PD-L1 mRNA expression **(C)**, MSI **(D)**, and TMB **(E)** across 33 types of cancer. The coefficient was evaluated by the Spearman analysis and the results were presented as radar maps (*p < 0.05; **p < 0.01; ***p < 0.001).

**FIGURE 7:**
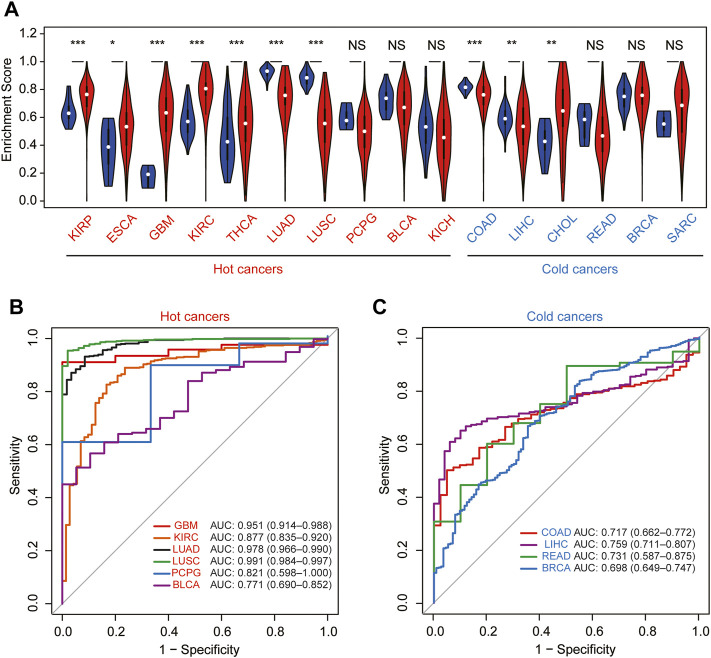
Diagnostic value of the module–gene of ferroptosis in pan-cancer. **(A)** Differential expression of the enrichment score between normal and tumor tissues in 16 types of cancer. Cancers were further divided into hot-cancers and cold-cancers based on the immune activation and presented in red and blue colors, respectively. *p < 0.05; **p < 0.01; and ***p < 0.001 was calculated using the Wilcoxon rank-sum test. The receiver operating characteristic curve (ROC) for the prediction of normal and tumor samples using the enrichment score in hot-cancers **(B)** and cold-cancers **(C)**.

To further explore whether the correlation of the module–gene with cancer immunity could be confirmed in pan-cancer, the mRNA expression of the module–gene in 33 types of cancer was obtained from the TCGA database. Consistent with SKCM, the enrichment score of the module–gene was positively correlated with the stromal score, immune score, ESTIMATE score, and infiltration of immune cells, but negatively related with tumor purity in nearly all types of cancer (Figures 6A,B). Moreover, positive correlations were also observed between the enrichment score and the mRNA expression of PD-L1 in most of cancers, except PCPG, LAML, and KICH (Figure 6C). On the contrary, negative correlations were found between the module–gene and MSI or TMB scores in nearly half of cancers (Figures 6D,E). As cancer cells may sensitize to ferroptosis differently based on the genetic background (Sosa et al., 2013; Spangler et al., 2016), we further explore the differential expression of the module–gene between normal and tumor tissues in 16 types of cancer. As expected, the module–gene was significantly increased in 70% of “hot-cancers” but was decreased in 50% of “cold-cancers,” further indicating the module–gene correlated with immune-activity cancers (Figure 7A). Moreover, the ROC curves showed the differential expressed module–gene had a great diagnostic value in “hot-cancers” and “cold-cancers” (Figure 7B). Overall, the correlation of the module–gene with cancer immunity was confirmed in the development of most cancers.

## Discussion

Ferroptosis, a novel procedure of cell death, was involved in both the activation and efficacy of immune cells. Considering melanoma response most effectively to immunotherapy, our current study first constructed and identified a key gene module of ferroptosis, which was closely related with the diagnosis and the prognosis of SKCM. Of note, the elevation of the module–gene was positively related with the immune activation and sensitively responds to anti-PD-1 treatment, indicating their role as a good biomarker for immunotherapy. More importantly, the correlation of the ferroptosis-related module and immune activation could be confirmed in the pan-cancer level.

More than 100 genes are involved in ferroptosis, resulting from the metabolic dysfunction through ROS generation, iron metabolism, and GSH synthesis (Hassannia et al., 2019). However, various ferroptosis-related genes are expressed in cancer (Zhang et al., 2020). In this study, we first constructed a co-expression network and identified the turquoise module as the key signature of ferroptosis-related genes. Among turquoise module–gene, there were inter-correlations with each other at both mRNA and protein levels. Previous studies have been mainly focused on the prognosis value of ferroptosis-related genes in melanoma using the public database (Jin et al., 2021; Luo and Ma, 2021; Ping et al., 2022). However, in our study, we highlighted the strong connection between ferroptosis and landscape tumor immunity, which might contribute to the ferroptosis-related prognostic value. Moreover, we found the important module–gene was closely related to clinical immunotherapy. Our study suggested that ferroptosis could impact the clinical outcome though immune activation. Interestingly, these results could be confirmed in the pan-cancer level. For example, the elevated module–gene was found in 70% of “hot-cancers,” such as KIRP, ESCA, GBM, HNSC, KIRC, and THCA, which are defined as the high immune activity, whereas decreasing of the module–gene was observed in half of “cold-cancers,” indicating low immune activity cancers. Therefore, this ferroptosis module could act as the sensitive marker for the diagnosis of most pan-cancers, which was dependent on the immunity activities.

Increasing experimental evidence indicated the interaction of ferroptosis and immunity in carcinogenesis. First, ferroptotic cells could activate immune cells, and 15-lipoxygenase mediated lipid oxidation regulation, the maturation of dendritic cells, and the activation of the adaptive immune system (Rothe et al., 2015). Evidence also proved that macrophages could be recruited and engulf the ferroptotic cells (Klöditz and Fadeel, 2019). Reversely, ROS production, which activates ferroptosis, was required for NK cell-mediated cell death (Roder et al., 1982; Ramstedt et al., 1984). On the other hand, CD8^+^ T cells could induce lipid peroxidation and ferroptosis during immunotherapy (Wang et al., 2019). Specifically, interferon gamma released from CD8^+^ T cells restrain the synthesis of GSH through downregulating SLC3A2 and SLC7A11, therefore leading to the accumulation of peroxidated phospholipid and ferroptosis in tumor cells. In our study, we further confirmed this strong correlation of ferroptosis and immunity, and provide potential targets for future research studies. In short, we established a gene–module bridging ferroptosis and immunity by multiple public clinical databases, thereby impacting the clinical outcomes of SKCM patients. However, the limitation of the current study is the lack of information about the resource of ferroptosis gene in the sub-cellular level. Further analysis using scRNA-seq and spatial transcriptomics could provide more evidence. In addition, the underlying mechanism of the effect of ferroptosis on cell immunity needs further validation by *in vivo* and *in vitro* experiments. For instance, the immune cell activity and quantity could be measured by co-culture with SKCM cell lines or mice models with gain- or loss-of-function on the ferroptosis module gene.

Since the response of immunotherapy differs among patients and cancers, the predictive biomarkers for immunotherapy have been studied. The elevated expression of PD-L1 in cancer cells was recognized as a consequence of tumor immune escape and a biomarker for the prediction of immune checkpoint inhibitors (Chemnitz et al., 2004; Jiang et al., 2019). Of note, both the upregulation of PD-L1 and the promotion of ferroptosis were driven by CD8^+^ T cells released IFNγ (Spranger et al., 2013; Wang et al., 2019), which foreshowed that T cell-mediated ferroptosis might be involved with the phenotypic alteration in cancer cells. Here, we found a module–gene of ferroptosis was positively correlated with the mRNA expression of PD-L1 in pan-cancer, indicating these genes might participate in the regulation of PD-L1. In addition, preclinical studies found that neoantigens produced by tumors were primary drivers of antitumor immune responses (Castle et al., 2012; Matsushita et al., 2012). Based on these studies, patients with high TMB and MSI scores were considered more responsive to immunotherapy strategies (Goodman et al., 2019). However, we found correlation between the module–gene and TMB or MSI scores is dependent on tumors, indicating ferroptosis in cancer might be triggered independent from genetic mutational burdens.

In summary, we identified a critical module of ferroptosis, which has a strong correlation with the diagnosis, prognosis, and tumor immune activity in melanoma. In particular, the module was positively related with the expression of PDL1 and sensitively responds to anti-PD-1 treatment, indicating the role as a good biomarker for immunotherapy. We further confirmed their correlation with tumor immunity in pan-cancer. Our studies highlighted that ferroptosis was involved in antitumor immunity and might provide a prospective gene target for immunotherapy.

## Data Availability

The datasets presented in this study can be found in online repositories. The names of the repository/repositories and accession number(s) can be found in the article/Supplementary Material.
