# Supplementary figures and images for: Ferroptosis Associates With Diagnosis and Prognosis by Promoting Antitumor Immune Response in Melanoma
Source: Front Cell Dev Biol. 2022 Jul 8;10:915198. doi: 10.3389/fcell.2022.915198 (PMC9304890; doi:10.3389/fcell.2022.915198)

## Slide 1
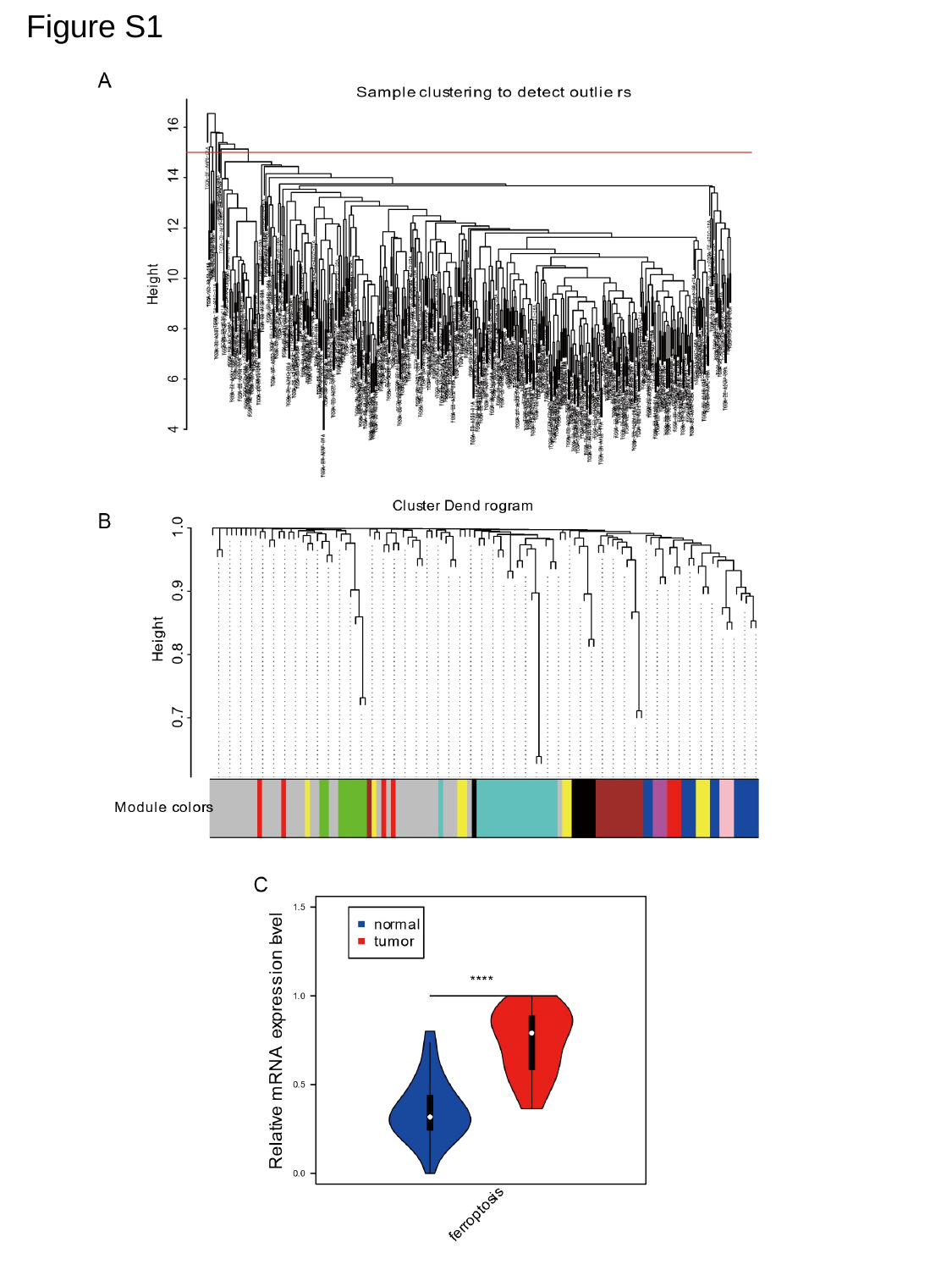

Figure S1

## Slide 2
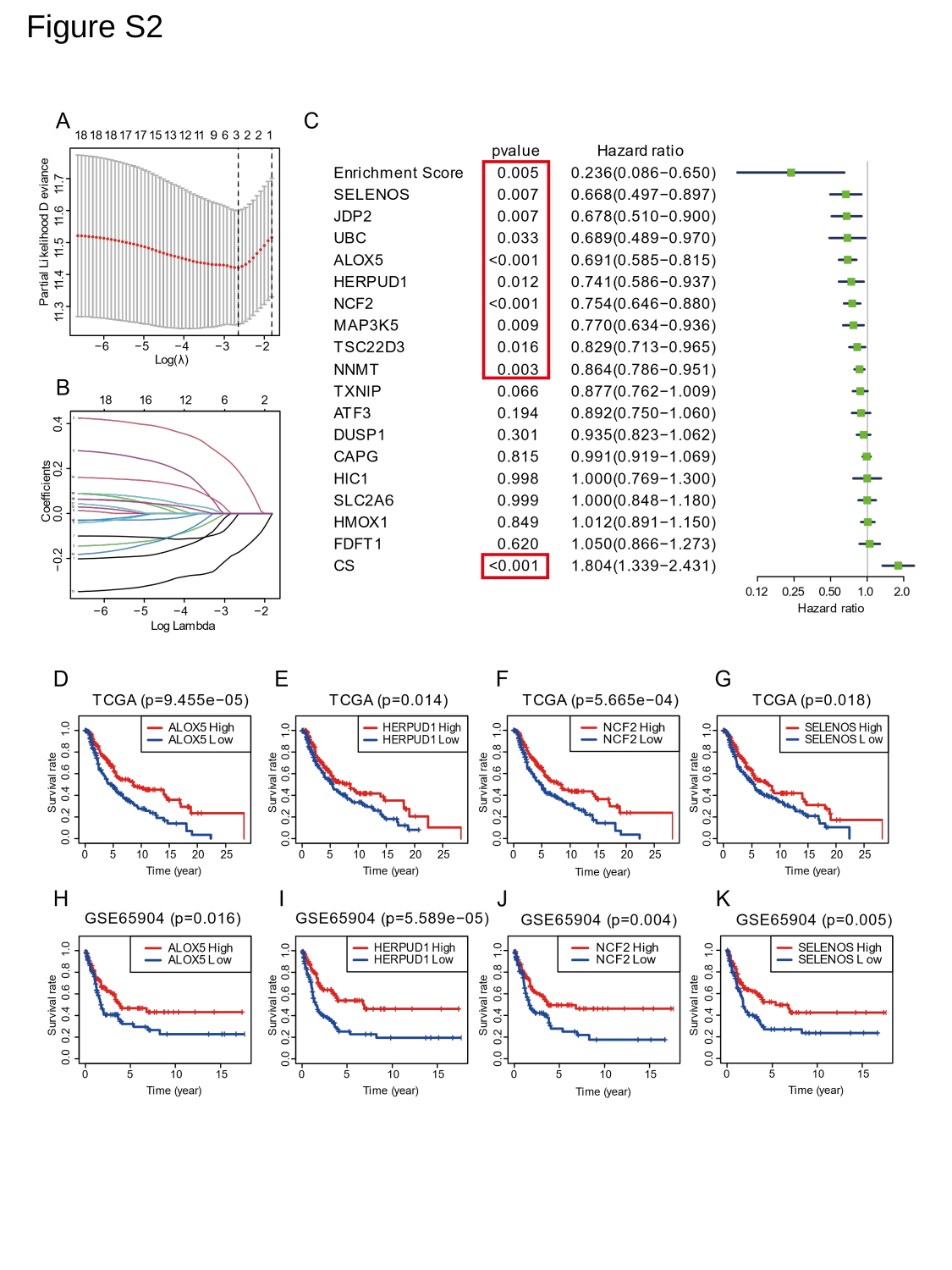

Figure S2

Supplement: Supplementary file 3 [file Presentation1.PPTX]
