# Supplementary material for: Ferroptosis Associates With Diagnosis and Prognosis by Promoting Antitumor Immune Response in Melanoma
Source: Front Cell Dev Biol. 2022 Jul 8;10:915198. doi: 10.3389/fcell.2022.915198 (PMC9304890; doi:10.3389/fcell.2022.915198)
Supplement: Supplementary file 4 [file Table5.DOCX]

**R code file**

**Figure 1a**

#if (!requireNamespace("BiocManager", quietly = TRUE))

# install.packages("BiocManager")

#BiocManager::install("AnnotationDbi")

#BiocManager::install("impute")

#BiocManager::install("GO.db")

#BiocManager::install("preprocessCore")

#site="https://mirrors.tuna.tsinghua.edu.cn/CRAN"

#install.packages(c("WGCNA", "stringr", "reshape2"), repos=site)

setwd("")

library(WGCNA)

rt=read.table("Fig1a_input1.txt",sep="\t",row.names=1,header=T,check.names=F,quote="!")

immune=read.table("Fig1a_input2.txt",sep="\t",header=T,check.names=F,row.names=1)

clinical=read.table("Fig1a_input3.txt",sep="\t",header=T,check.names=F,row.names=1)

score=read.table("Fig1a_input4.txt",sep="\t",header=T,check.names=F,row.names=1)

datExpr0=rt

datExpr = rt

gsg = goodSamplesGenes(datExpr0, verbose = 3)

gsg$allOK

if (!gsg$allOK)

{

if (sum(!gsg$goodGenes)>0)

printFlush(paste("Removing genes:", paste(names(datExpr0)[!gsg$goodGenes], collapse = ", ")));

if (sum(!gsg$goodSamples)>0)

printFlush(paste("Removing samples:", paste(rownames(datExpr0)[!gsg$goodSamples], collapse = ", ")));

datExpr0 = datExpr0[gsg$goodSamples, gsg$goodGenes]

}

if(T){

sampleTree = hclust(dist(datExpr0), method = "average")

pdf(file = "FigS1A.pdf", width = 12, height = 9)

par(cex = 0.6)

par(mar = c(0,4,2,0))

plot(sampleTree, main = "Sample clustering to detect outliers", sub="", xlab="", cex.lab = 1.5,

cex.axis = 1.5, cex.main = 2)

abline(h = 15, col = "red")

dev.off()

}

clust = cutreeStatic(sampleTree, cutHeight = 15, minSize = 0)

table(clust)

keepSamples = (clust==1)

datExpr = datExpr0[keepSamples, ]

write.table(datExpr,file="remained samples.txt.tsv",sep="\t",quote=F,col.names=T)

immune=t(immune)

remainedSamples = match(rownames(datExpr), rownames(immune))

remainedimmune = immune[remainedSamples,-31]

remainedimmune=t(remainedimmune)

str(remainedimmune)

write.table(remainedimmune,file="remainedimmune.txt.tsv",sep="\t",quote=T,col.names=T)

#remainedimmune=as.data.frame(remainedimmune)

remainedSamples = match(rownames(datExpr), rownames(clinical))

remainedclinical = clinical[remainedSamples,-31]

str(remainedclinical)

write.table(remainedclinical,file="remainedclinical.txt.tsv",sep="\t",quote=T,col.names=T)

remainedSamples = match(rownames(datExpr), rownames(score))

remainedscore = score[remainedSamples,-31]

str(remainedscore)

write.table(remainedscore,file="remainedscore.txt.tsv",sep="\t",quote=T,col.names=T)

group=sapply(strsplit(colnames(remainedimmune),"\\-"),"[",4)

group=sapply(strsplit(group,""),"[",1)

group=gsub("2","1",group)

remainedimmune=remainedimmune[,group==0]

#hierarchical cluster

hc = hclust(dist(t(remainedimmune)))

y=cutree(hc,2)

write.table(y,file="cluster.txt",sep="\t",quote=F,col.names=T)

powers = c(c(1:10), seq(from = 12, to=20, by=2))

sft = pickSoftThreshold(datExpr, powerVector = powers, verbose = 5)

if(T){

pdf(file = "Soft threshold.pdf", width = 18, height = 10)

par(mfrow = c(1,2))

cex1 = 0.9

plot(sft$fitIndices[,1], -sign(sft$fitIndices[,3])*sft$fitIndices[,2],

xlab="Soft Threshold (power)",

ylab="Scale Free Topology Model Fit,signed R^2",type="n",

main = paste("Scale independence"))

text(sft$fitIndices[,1], -sign(sft$fitIndices[,3])*sft$fitIndices[,2],

labels=powers,cex=cex1,col="red")

abline(h=0.90,col="red")

plot(sft$fitIndices[,1], sft$fitIndices[,5],

xlab="Soft Threshold (power)",ylab="Mean Connectivity", type="n",

main = paste("Mean connectivity"))

text(sft$fitIndices[,1], sft$fitIndices[,5], labels=powers, cex=cex1,col="red")

dev.off()

}

net = blockwiseModules(datExpr, power = 4,

TOMType = "unsigned", minModuleSize = 3,

reassignThreshold = 0, mergeCutHeight = 0.25,

numericLabels = TRUE, pamRespectsDendro = FALSE,

saveTOMs = TRUE,

saveTOMFileBase = "femaleMouseTOM",

verbose = 3)

table(net$colors)

mergedColors = labels2colors(net$colors)

if(T){

pdf(file = "FigS1B.pdf", width = 18, height = 10)

plotDendroAndColors(net$dendrograms[[1]], mergedColors[net$blockGenes[[1]]],

"Module colors",

dendroLabels = FALSE, hang = 0.03,

addGuide = TRUE, guideHang = 0.05)

dev.off()

}

moduleLabels = net$colors

moduleColors = labels2colors(net$colors)

MEs = net$MEs

geneTree = net$dendrograms[[1]]

write.table(net$colors,file="net$colors.txt",sep="\t",quote=F,col.names=F)

write.table(mergedColors,file="mergedColors.txt",sep="\t",quote=F,col.names=F)

save(MEs, moduleLabels, moduleColors, geneTree,

file = "wgcna.RData")

datTraits=remainedclinical

remainedSamples = match(rownames(datExpr), rownames(datTraits))

datTraits = datTraits[remainedSamples,-99]

datTraits=as.data.frame(datTraits)

nGenes = ncol(datExpr)

nSamples = nrow(datExpr)

MEs0 = moduleEigengenes(datExpr, moduleColors)$eigengenes

MEs = orderMEs(MEs0)

moduleTraitCor = cor(MEs, datTraits, use = "p")

moduleTraitPvalue = corPvalueStudent(moduleTraitCor, nSamples)

if(T){

pdf(file = "Fig1A.pdf", width = 18, height = 10)

textMatrix = paste(signif(moduleTraitCor, 2), "\n(",

signif(moduleTraitPvalue, 1), ")", sep = "")

dim(textMatrix) = dim(moduleTraitCor)

par(mar = c(6, 8.5, 3, 3))

labeledHeatmap(Matrix = moduleTraitCor,

xLabels = names(datTraits),

yLabels = names(MEs),

ySymbols = names(MEs),

colorLabels = FALSE,

colors = greenWhiteRed(50),

textMatrix = textMatrix,

setStdMargins = FALSE,

cex.text = 1,

zlim = c(-1,1),

main = paste("Module-trait relationships"))

dev.off()

}

**Figure 1b**

setwd("")

rt=read.table("Fig1b_input.txt.tsv",sep="\t",header=T,row.names=1,check.names=F)

rt=t(rt)

library(pheatmap)

library(ComplexHeatmap)

rt=as.matrix(rt)

library(corrplot)

res1 <- cor.mtest(rt, conf.level = 0.95)

pdf("Fig1b.pdf",height=8,width=8)

corrplot(corr=cor(rt),

method = "circle",

order = "hclust",

type="upper",

tl.col="black",

number.cex = 0.6,

col=colorRampPalette(c("blue", "white", "red"))(50),

title="correlation of genes in mRNA level",

mar=c(2,2,3,2),

diag=F,

p.mat = res1$p, sig.level = 0.05,

)

dev.off()

**Figure 1d**

library(vioplot)

setwd("C:\\Users\\Administrator\\Desktop\\test")

files=grep(".txt",dir(),value = T)

for (i in files) {

rt=read.table(i,sep="\t",header=T,row.names=1,check.names=F)

rt=data.frame(rt)

genename=sapply(strsplit(i,".txt"),"[",1)

group=levels(factor(rt$CancerType))

group=c("SLC2A6","HIC1","ATF3","ALOX5","NNMT","NCF2","HMOX1","SELENOS","CAPG","DUSP1","HERPUD1","UBC","JDP2","MAP3K5","CS","FDFT1","TSC22D3")

ymax=max(rt[,1])

outfile=paste0(genename,".pdf")

pdf(outfile,height=8,width=20)

par(las=1,mar=c(10,6,3,3))

x=c(1:19)

y=c(1:19)

plot(x,y,

xlim=c(0,36),ylim=c(min(rt[,1]),max(rt[,1])+0.5),

main="",xlab="", ylab="relative mRNA expression levels",cex.lab = 2,

pch=21,

col="white",

xaxt="n")

text(seq(1,37,2.22),-ymax/10,xpd = NA,labels=group,cex = 1.7,srt = 45,pos=2)

text(19,ymax*1.15,xpd = NA,labels="GSE98384",cex = 2,pos=2)

legend(1,ymax*1, c('normal','tumor'), col = c("blue", "red"),text.col ='black', pch = c(15, 15 ), bg ='white',pt.cex=1.6,cex=1.6)

for (j in group) {

rt1=rt[rt[,"CancerType"]==j,]

count=which(group==j)

normal=rt1[rt1[,"Type"]=="normal",]

normal=as.data.frame(normal)

tumor= rt1[rt1[,"Type"]=="tumor",]

tumor=as.data.frame(tumor)

normalData=as.numeric(normal[,1])

tumorData=as.numeric(tumor[,1])

vioplot(normalData,at=2.2*(count-1),lty=1,add = T,col = 'blue')

vioplot(tumorData,at=2.2*(count-1)+1,lty=1,add = T,col = 'red')

wilcoxTest=wilcox.test(normalData,tumorData,exact=FALSE)

p=round(wilcoxTest$p.value,3)

mx=max(c(normalData,tumorData))

lines(c(x=2.2*(count-1)+0.2,x=2.2*(count-1)+0.8),c(mx,mx))

text(x=2.2*(count-1)+0.5,y=mx+0.5,labels=ifelse(p<0.001,paste0("****"),ifelse(p<0.01,paste0("***"),paste0("NS"))),cex = 1.6)

}

dev.off()

}

**Figure 1e**

library(dplyr)

library(pROC)

setwd("")

rt <- read.table("Fig1e_input.txt",header=T,sep="\t",check.names=F)

rt <- rt %>% select(ferroptosis,Type,CancerType)

cancertype <- levels(factor(rt$CancerType))

for (i in cancertype) {

data <- rt[rt$CancerType==i,]

roc1 <- roc(data$Type, data$pyroptosis,ci=TRUE)

levels(data$Type) <- c(`Normal`=0,`Tumor`=1)

# ci(roc1)

# ci(roc1, of="auc")

# ci.auc(roc1)

# roc2 <- roc(rt$GROUP, rt$`Inflammatory Caspases`)

# roc3 <- roc(rt$GROUP, rt$`Gasdermin Family`)

# roc4 <- roc(rt$GROUP, rt$`Pro-inflammatory Cytokines`)

auc(roc1)

ci(roc1,of="auc")

# auc(roc2)

# auc(roc3)

# auc(roc4)

outfile <- paste0("ROC_",i,".pdf")

pdf(file=outfile,width=6,height=6)

# par(oma=c(0.5,1,0,1),font.lab=1.5,font.axis=1.5)

plot.roc(roc1, col="red",print.auc=T,print.auc.pattern=NULL,legacy.axes=T)

# plot(roc1$specificities, roc1$sensitivities, type="l", xlim=c(0,1), ylim=c(0,1),col='red',

# xlab="specificity", ylab="sensitivity",

# main=paste("ROC curve (", "AUC = ",round(roc1$auc,3),")"),

# lwd = 2, cex.main=1.3, cex.lab=1.2, cex.axis=1.2, font=1.2)

# plot.roc(roc2, add=TRUE, col="blue")

# plot.roc(roc3, add=TRUE, col="green")

# plot.roc(roc4, add=TRUE, col="orange")

par(mar=c(0,0,0,2),xpd = T,cex.axis=1.6)

legend("topright",

legend="lasso(ssgesa)",

col=c("red","blue","green","orange"),

title=i,

lty=1,lwd=2,xpd=TRUE)

print(plot)

dev.off()

}

**Figure 2a**

#install.packages("survival")

setwd("")

library(survival)

pFilter=1

rt=read.table("Fig2a_input.txt",header=T,sep="\t",check.names=F)

rt$futime=rt$futime/365

outTab=data.frame()

for(gene in colnames(rt[,4:ncol(rt)])){

if(sd(rt[,gene])==0){

next}

a=rt[,gene]<=median(rt[,gene])

diff=survdiff(Surv(futime, fustat) ~a,data = rt)

pValue=1-pchisq(diff$chisq,df=1)

outTab=rbind(outTab,cbind(gene=gene,pvalue=pValue))

fit <- survfit(Surv(futime, fustat) ~ a, data = rt)

summary(fit)

if(pValue<pFilter){

if(pValue<0.001){

pValue=signif(pValue,4)

pValue=format(pValue, scientific = TRUE)

}else{

pValue=round(pValue,3)

}

pdf(file=paste(gene,".survival.pdf",sep=""),

width = 5.5,

height =5,

)

plot(fit,

lwd=2,

col=c("red","blue"),

xlab="Time (year)",

mark.time=T,

ylab="Survival rate",

ylim=c(0,1.09),

main=paste("Survival curve (p=", pValue ,")",sep=""))

legend("topright",

c(paste(gene," high expression",sep=""),

paste(gene," low expression",sep="") ),

lwd=2,

col=c("red","blue"))

dev.off()

}

}

write.table(outTab,file="survival.xls",sep="\t",row.names=F,quote=F)

**Figure 2b**

#install.packages("survival")

setwd("")

library(survival)

pFilter=1

rt=read.table("Fig2b_input.txt",header=T,sep="\t",check.names=F)

rt$futime=rt$futime/365

outTab=data.frame()

for(gene in colnames(rt[,4:ncol(rt)])){

if(sd(rt[,gene])==0){

next}

a=rt[,gene]<=median(rt[,gene])

diff=survdiff(Surv(futime, fustat) ~a,data = rt)

pValue=1-pchisq(diff$chisq,df=1)

outTab=rbind(outTab,cbind(gene=gene,pvalue=pValue))

fit <- survfit(Surv(futime, fustat) ~ a, data = rt)

summary(fit)

if(pValue<pFilter){

if(pValue<0.001){

pValue=signif(pValue,4)

pValue=format(pValue, scientific = TRUE)

}else{

pValue=round(pValue,3)

}

pdf(file=paste(gene,".survival.pdf",sep=""),

width = 5.5,

height =5,

)

plot(fit,

lwd=2,

col=c("red","blue"),

xlab="Time (year)",

mark.time=T,

ylab="Survival rate",

ylim=c(0,1.09),

main=paste("Survival curve (p=", pValue ,")",sep=""))

legend("topright",

c(paste(gene," high expression",sep=""),

paste(gene," low expression",sep="") ),

lwd=2,

col=c("red","blue"))

dev.off()

}

}

write.table(outTab,file="survival.xls",sep="\t",row.names=F,quote=F)

**Figuer 2c**

#install.packages('survival')

#install.packages('forestplot')

setwd("C:")

library(survival)

library(forestplot)

options(forestplot_new_page = FALSE)

clrs <- fpColors(box="green",line="darkblue", summary="royalblue")

rt=read.table("Fig2c_input.txt",header=T,sep="\t",check.names=F,row.names=1)

outTab=data.frame()

for(i in colnames(rt[,3:ncol(rt)])){

cox <- coxph(Surv(futime, fustat) ~ rt[,i], data = rt)

coxSummary = summary(cox)

coxP=coxSummary$coefficients[,"Pr(>|z|)"]

outTab=rbind(outTab,

cbind(id=i,

HR=coxSummary$conf.int[,"exp(coef)"],

HR.95L=coxSummary$conf.int[,"lower .95"],

HR.95H=coxSummary$conf.int[,"upper .95"],

pvalue=coxSummary$coefficients[,"Pr(>|z|)"])

)

}

write.table(outTab,file="uniCox.xls",sep="\t",row.names=F,quote=F)

rt=read.table("uniCox.xls",header=T,sep="\t",row.names=1,check.names=F)

data=as.matrix(rt)

HR=data[,1:3]

hr=sprintf("%.3f",HR[,"HR"])

hrLow=sprintf("%.3f",HR[,"HR.95L"])

hrHigh=sprintf("%.3f",HR[,"HR.95H"])

pVal=data[,"pvalue"]

pVal=ifelse(pVal<0.001, "<0.001", sprintf("%.3f", pVal))

tabletext <-

list(c(NA, rownames(HR)),

append("pvalue", pVal),

append("Hazard ratio",paste0(hr,"(",hrLow,"-",hrHigh,")")) )

pdf(file="Fig2c.pdf",

width = 6,

height = 4,

)

forestplot(tabletext,

rbind(rep(NA, 3), HR),

col=clrs,

graphwidth=unit(50, "mm"),

xlog=T,

lwd.ci=2,

boxsize=0.3,

xlab="Hazard ratio",

title="SKCM"

)

dev.off()

**Figure 2d**

#install.packages('survival')

#install.packages('forestplot')

library(survival)

library(forestplot)

options(forestplot_new_page = FALSE)

clrs <- fpColors(box="red",line="darkblue", summary="royalblue")

setwd("")

rt=read.table("Fig2d_input.txt",header=T,sep="\t",check.names=F,row.names=1)

multiCox=coxph(Surv(futime, fustat) ~ ., data = rt)

multiCoxSum=summary(multiCox)

outTab=data.frame()

outTab=cbind(

HR=multiCoxSum$conf.int[,"exp(coef)"],

HR.95L=multiCoxSum$conf.int[,"lower .95"],

HR.95H=multiCoxSum$conf.int[,"upper .95"],

pvalue=multiCoxSum$coefficients[,"Pr(>|z|)"])

outTab=cbind(id=row.names(outTab),outTab)

write.table(outTab,file="multiCox.xls",sep="\t",row.names=F,quote=F)

rt=read.table("multiCox.xls",header=T,sep="\t",row.names=1,check.names=F)

data=as.matrix(rt)

HR=data[,1:3]

hr=sprintf("%.3f",HR[,"HR"])

hrLow=sprintf("%.3f",HR[,"HR.95L"])

hrHigh=sprintf("%.3f",HR[,"HR.95H"])

pVal=data[,"pvalue"]

pVal=ifelse(pVal<0.001, "<0.001", sprintf("%.3f", pVal))

tabletext <-

list(c(NA, rownames(HR)),

append("pvalue", pVal),

append("Hazard ratio",paste0(hr,"(",hrLow,"-",hrHigh,")")) )

pdf(file="Fig2d.pdf",

width = 6,

height = 4,

)

forestplot(tabletext,

rbind(rep(NA, 3), HR),

col=clrs,

graphwidth=unit(50, "mm"),

xlog=T,

lwd.ci=2,

boxsize=0.3,

xlab="Hazard ratio",

title="SKCM"

)

dev.off()

**Figure 2e**

#install.packages("survival")

setwd("")

library(survival)

rt=read.table("Fig2e_input.txt",header=T,sep="\t")

diff=survdiff(Surv(futime, fustat) ~risk,data = rt)

pValue=1-pchisq(diff$chisq,df=1)

pValue=round(pValue,5)

fit <- survfit(Surv(futime, fustat) ~ risk, data = rt)

summary(fit)

pdf(file="Fig2e.pdf")

plot(fit, lty = 2:3,col=c("red","blue"),xlab="time (year)",ylab="survival rate",

main=paste("survival curve (p=", pValue ,")",sep=""),mark.time=T)

legend("topright", c("high risk", "low risk"), lty = 2:3, col=c("red","blue"))

dev.off()

**Figure 2f**

library(survivalROC)

setwd("")

rt=read.table("Fig2f_input.txt",header=T,sep="\t",check.names=F,row.names=1)

pdf(file="Fig2f.pdf")

par(oma=c(0.5,1,0,1),font.lab=1.5,font.axis=1.5)

roc=survivalROC(Stime=rt$futime, status=rt$fustat, marker = rt$riskScore,

predict.time =10, method="KM")

plot(roc$FP, roc$TP, type="l", xlim=c(0,1), ylim=c(0,1),col='red',

xlab="False positive rate", ylab="True positive rate",

main=paste("ROC curve (", "AUC = ",round(roc$AUC,3),")"),

lwd = 2, cex.main=1.3, cex.lab=1.2, cex.axis=1.2, font=1.2)

abline(0,1)

dev.off()

**Figure 3a**

#install.packages("pheatmap")

#install.packages('sparcl')

library(sparcl)

library(pheatmap)

setwd("")

data=read.table("Fig3a_input1",sep="\t",header=T,check.names=F,row.names=1)

data=t(data)

group=sapply(strsplit(colnames(data),"\\-"),"[",4)

group=sapply(strsplit(group,""),"[",1)

group=gsub("2","1",group)

data=data[,group==0]

#hierarchical cluster

hc = hclust(dist(t(data)))

y=cutree(hc,2)

write.table(y,file="cluster.txt",sep="\t",quote=F,col.names=F)

pdf(file="hclust.pdf",width=50,height=20)

ColorDendrogram(hc, y = y, labels = names(y), branchlength = 0.3,xlab=" ",sub=" ",main = " ")

dev.off()

rt=read.table("Fig3a_input1",sep="\t",header=T,row.names=1,check.names=F) #??ȡ?ļ?

rt=t(rt)

Type=read.table("cluster.txt",sep="\t",check.names=F,row.names=1,header=F)

rt=rt[,row.names(Type)]

ferroptosis=read.table("Fig3a_input2.txt",sep="\t",check.names=F,row.names=1,header=T)

ferroptosis=ferroptosis[row.names(Type),]

score=read.table("Fig3a_input3.txt",sep="\t",check.names=F,row.names=1,header=T)

score=score[row.names(Type),]

colnames(Type)=c("cluster","Subtype")

cluster=cbind(Type,ferroptosis=ferroptosis[,1],score[,1:4])

annotation_col=colnames(cluster)

cluster=cluster[,-1]

cluster1 <- cluster[,c(6,5,4,3,2,1)]

Type=Type[order(Type$Subtype),]

rt=rt[,row.names(Type)]

pdf("Fig3a.pdf",height=5.5,width=9)

pheatmap(rt, annotation=cluster1,

color = colorRampPalette(c("blue", "white", "red"))(50),

cluster_cols =F,

fontsize=8,

fontsize_row=8,

scale="row",

main="SKCM",

show_colnames=F,

fontsize_col=3)

dev.off()

**Figure 3b-d**

#install.packages("ggplot2")

#install.packages("ggpubr")

#install.packages("ggExtra")

library(ggplot2)

library(ggpubr)

library(ggExtra)

setwd("")

pFilter=10

exp=read.table("Fig3b-d_input1.txt", header=T,sep="\t",row.names=1,check.names=F)

gene=colnames(exp)[1]

TME=read.table("Fig3b-d_input2.txt", header=T,sep="\t",row.names=1,check.names=F)

group=sapply(strsplit(row.names(exp),"\\-"),"[",4)

group=sapply(strsplit(group,""),"[",1)

group=gsub("2","1",group)

exp=exp[group==0,]

sameSample=intersect(row.names(TME),row.names(exp))

TME=TME[sameSample,]

exp=exp[sameSample,]

y=as.numeric(exp[,1])

for(j in colnames(TME)[1:4]){

x=as.numeric(TME[,j])

df1=as.data.frame(cbind(x,y))

corT=cor.test(x,y,method="spearman")

cor=corT$estimate

pValue=corT$p.value

p1=ggplot(df1, aes(x, y)) +

xlab(j)+ylab(gene)+

ggtitle(paste("corrlation "))+theme(title=element_text(size=10))+

geom_point()+ geom_smooth(method="lm") + theme_bw()+

stat_cor(method = 'spearman', aes(x =x, y =y),label.y = 1.25)

p2=ggMarginal(p1, type = "density", xparams = list(fill = "orange"),yparams = list(fill = "blue"))

if(pValue<pFilter){

pdf(file=paste0("Fig3","_",j,".pdf"),width=5,height=5)

print(p2)

dev.off()

}

}

dev.off()

**Figure 4a**

library(vioplot)

setwd("")

files=grep(".txt",dir(),value = T)

for (i in files) {

rt=read.table(i,sep="\t",header=T,row.names=1,check.names=F)

rt=data.frame(rt)

genename=sapply(strsplit(i,".txt"),"[",1)

group=levels(factor(rt$CancerType))

group=c( "SLC2A6","ATF3","JDP2","HERPUD1","NNMT","MAP3K5","FDFT1","ALOX5","HMOX1","NCF2","TXNIP","TSC22D3","SELENOS","DUSP1","UBC","HIC1","CAPG","CS")

ymax=max(rt[,1])

outfile=paste0(Fig4a,".pdf")

pdf(outfile,height=8,width=20)

par(las=1,mar=c(10,6,3,3))

x=c(1:19)

y=c(1:19)

plot(x,y,

xlim=c(0,38),ylim=c(min(rt[,1]),max(rt[,1])+0.5),

main="",xlab="", ylab="relative mRNA expression levels",cex.lab = 2,

pch=21,

col="white",

xaxt="n")

text(seq(1,39,2.22),3,xpd = NA,labels=group,cex = 1.7,srt = 45,pos=2)

text(19,ymax*1.1,xpd = NA,labels="GSE91061",cex = 2,pos=2)

legend(0.5,ymax*1.04, c('responser-pre','responser-on'), col = c("blue","red"),text.col ='black', pch = c(15, 15 ), bg ='white',pt.cex=1.6,cex=1.6)

for (j in group) {

rt1=rt[rt[,"CancerType"]==j,]

count=which(group==j)

respre=rt1[rt1[,"Type"]=="res-pre",]

respre=as.data.frame(respre)

reson= rt1[rt1[,"Type"]=="res-on",]

reson=as.data.frame(reson)

respreData=as.numeric(respre[,1])

resonData=as.numeric(reson[,1])

vioplot(respreData,at=2.2*(count-1),lty=1,add = T,col = 'blue')

vioplot(resonData,at=2.2*(count-1)+1,lty=1,add = T,col = 'red')

wilcoxTest=wilcox.test(respreData,resonData,exact=FALSE,paired = TRUE)

p=round(wilcoxTest$p.value,3)

mx=max(c(respreData,resonData))

lines(c(x=2.2*(count-1)+0.2,x=2.2*(count-1)+0.8),c(mx,mx))

text(x=2.2*(count-1)+0.5,y=mx+0.5,labels=ifelse(p<0.001,paste0("***"),ifelse(p<0.01,paste0("**"),ifelse(p<0.05,paste0("*"),paste0("NS")))),cex = 2)

#print(vioplot)

}

dev.off()

}

**Figure 4b**

library(vioplot)

setwd("")

files=grep(".txt",dir(),value = T)

for (i in files) {

rt=read.table(i,sep="\t",header=T,row.names=1,check.names=F)

rt=data.frame(rt)

genename=sapply(strsplit(i,".txt"),"[",1)

group=levels(factor(rt$CancerType))

group=c( "SLC2A6","ATF3","JDP2","HERPUD1","NNMT","MAP3K5","FDFT1","ALOX5","HMOX1","NCF2","TXNIP","TSC22D3","SELENOS","DUSP1","UBC","HIC1","CAPG","CS")

ymax=max(rt[,1])

outfile=paste0(Fig4b,".pdf")

pdf(outfile,height=8,width=20)

par(las=1,mar=c(10,6,3,3))

x=c(1:19)

y=c(1:19)

plot(x,y,

xlim=c(0,38),ylim=c(min(rt[,1]),max(rt[,1])+0.5),

main="",xlab="", ylab="relative mRNA expression levels",cex.lab = 2,

pch=21,

col="white",

xaxt="n")

text(seq(1,39,2.22),-ymax/40,xpd = NA,labels=group,cex = 1.7,srt = 45,pos=2)

text(19,ymax*1.1,xpd = NA,labels="GSE91061",cex = 2,pos=2)

legend(1,ymax*1.05, c('nonresponser-pre','nonresponser-on'), col = c("yellow","purple"),text.col ='black', pch = c(15, 15 ), bg ='white',pt.cex=1.6,cex=1.6)

for (j in group) {

rt1=rt[rt[,"CancerType"]==j,]

count=which(group==j)

nonrespre=rt1[rt1[,"Type"]=="nonres-pre",]

nonrespre=as.data.frame(nonrespre)

nonreson= rt1[rt1[,"Type"]=="nonres-on",]

nonreson=as.data.frame(nonreson)

nonrespreData=as.numeric(nonrespre[,1])

nonresonData=as.numeric(nonreson[,1])

vioplot(nonrespreData,at=2.2*(count-1),lty=1,add = T,col = 'yellow')

vioplot(nonresonData,at=2.2*(count-1)+1,lty=1,add = T,col = 'purple')

wilcoxTest=wilcox.test(nonrespreData,nonresonData,exact=FALSE)

p=round(wilcoxTest$p.value,3)

mx=max(c(nonrespreData,nonresonData))

lines(c(x=2.2*(count-1)+0.2,x=2.2*(count-1)+0.8),c(mx,mx))

text(x=2.2*(count-1)+0.5,y=mx+0.6,labels=ifelse(p<0.001,paste0("***"),ifelse(p<0.01,paste0("**"),ifelse(p<0.05,paste0("*"),paste0("NS")))),cex = 2)

#print(vioplot)

}

dev.off()

}

**Figure 5a**

library(ggplot2)

library(ggpubr)

library(ggExtra)

setwd("")

pFilter=0.001

exp=read.table("Fig5a_input1.txt", header=T,sep="\t",row.names=1,check.names=F)

gene=colnames(exp)[1]

TME=read.table("Fig5a_input2.txt", header=T,sep="\t",row.names=1,check.names=F)

group=sapply(strsplit(row.names(exp),"\\-"),"[",4)

group=sapply(strsplit(group,""),"[",1)

group=gsub("2","1",group)

exp=exp[group==0,]

sameSample=intersect(row.names(TME),row.names(exp))

TME=TME[sameSample,]

exp=exp[sameSample,]

outTab=data.frame()

outTab2=data.frame()

cancers <- levels(factor(exp[,"CancerType"]))

for(i in cancers){

exp1=exp[(exp[,"CancerType"]==i),]

TME1=TME[(TME[,"CancerType"]==i),]

y=as.numeric(exp1[,1])

outVector=data.frame(i,gene)

outVector2=data.frame(i,gene)

for(j in colnames(TME1)[1:4]){

x=as.numeric(TME1[,j])

if(sd(x)>0.01){

df1=as.data.frame(cbind(x,y))

corT=cor.test(x,y,method="spearman")

cor=corT$estimate

pValue=corT$p.value

outVector=cbind(outVector,pValue)

outVector2=cbind(outVector2,cor)

}

else{

outVector=cbind(outVector,pValue=1)

outVector2=cbind(outVector2,cor=0)

}

}

outTab=rbind(outTab,outVector)

outTab2=rbind(outTab2,outVector2)

}

colNames=c("CancerType","Gene",colnames(TME)[1:4])

colnames(outTab)=colNames

colnames(outTab2)=colNames

write.table(outTab,file="estimatepvalue.txt",sep="\t",row.names=F,quote=F)

write.table(outTab2,file="estimatecor.txt",sep="\t",row.names=F,quote=F)

cor=read.table("estimatecor.txt",header = T,sep = "\t",check.names = F,row.names = 1)

p=read.table("estimatepvalue.txt",header = T,sep = "\t",check.names = F,row.names = 1)

cor <- cor[,-1]

cor <- t(cor)

p<- p[,-1]

p <- t(p)

library(pheatmap)

pdf("Fig5a.pdf",width = 12,height = 6)

pheatmap(cor,

color = colorRampPalette(c("#008800", "white", "#AB221F"))(100),

cluster_rows =F,

scale="none",

fontsize = 15,

fontsize_row=15,

main="Fig5a",

display_numbers = matrix(ifelse(p< 0.01, "*", ""), nrow(p)))

dev.off()

write.table(cor,file="cor.txt",sep="\t",quote=F,col.names=F)

write.table(p,file="cor.txt",sep="\t",quote=F,col.names=F)

**Figure 5b**

setwd("")

exp=read.table("Fig5b_input1.txt", header=T,sep="\t",row.names=1,check.names=F)

IMM=read.table("Fig5b_input2.txt", header=T,sep="\t",row.names=1,check.names=F)

group=sapply(strsplit(row.names(exp),"\\-"),"[",4)

group=sapply(strsplit(group,""),"[",1)

group=gsub("2","1",group)

exp=exp[group==0,]

sameSample=intersect(row.names(IMM),row.names(exp))

IMM=IMM[sameSample,]

exp=exp[sameSample,]

cancers=levels(factor(exp[,"CancerType"]))

outTab=data.frame()

outTab2=data.frame()

outTab3=data.frame()

for(j in 1:(ncol(IMM)-2)){

for(i in cancers){

exp1=exp[(exp[,"CancerType"]==i),]

IMM1=IMM[(IMM[,"CancerType"]==i),]

x=as.numeric(IMM1[,j])

y=as.numeric(exp1[,1])

corT=cor.test(x,y,method="spearman",exact = F)

cor=corT$estimate

pValue=corT$p.value

sig=ifelse(pValue<0.001,"***",ifelse(pValue<0.01,"**",ifelse(pValue<0.05,"*"," ")))

immuneset=colnames(IMM)[j]

outTab=rbind(outTab,cbind(CancerType=i,cor=cor,pValue=pValue,sig,immuneset=immuneset))

}

}

write.table(outTab,file="cor.txt",sep="\t",row.names=F,quote=F)

library(dplyr)

IMM=read.table("Fig5b_input3.txt",sep = "\t")

IMM=as.character(IMM$V1)

data=read.table("cor.txt",sep = "\t",header = T,check.names = F)

CancerType=as.vector(data[1:33,"CancerType"])

outab=data.frame(CancerType=CancerType)

for (i in IMM) {

rt=data[data$immuneset==i,1:2]

colnames(rt)=c("CancerType",i)

outab=full_join(outab,rt,by="CancerType")

}

pdata=data[,-2]

CancerType=as.vector(data[1:33,"CancerType"])

ptab=data.frame(CancerType=CancerType)

for (i in IMM) {

rt=pdata[pdata$immuneset==i,1:2]

colnames(rt)=c("CancerType",i)

ptab=full_join(ptab,rt,by="CancerType")

}

rownames(outab)=outab[,1]

Colname=read.table("Fig5b_input4.txt",sep = "\t")

Colname=as.character(Colname$V1)

outab=outab[,Colname]

rownames(ptab)=ptab[,1]

ptab=ptab[,-1]

Colname=read.table("Fig5b_input4.txt",sep = "\t")

Colname=as.character(Colname$V1)

ptab=ptab[,Colname]

cancerType=read.table("Fig5b_input5.txt",sep="\t",header=T,row.names=1,check.names=F)

Cancerorder=read.table("Fig5b_input6.txt",sep="\t",header=T,row.names=1,check.names=F)

library(pheatmap)

outpdf="Fig5b.pdf"

pdf(outpdf,width = 12,height = 9)

outab=t(outab)

ptab=t(ptab)

outab=outab[,row.names(Cancerorder)]

ann_colors = list(

'cancertype' = c('cold' = "navy", 'hot' = "firebrick3")

)

bk <- c(seq(floor(min(outab)),-0.1,by=0.01),seq(0,ceiling(max(outab)),by=0.01))

pheatmap(outab,annotation_colors = ann_colors,

color = c(colorRampPalette(colors = c("blue","white"))(length(bk)/2),colorRampPalette(colors = c("white","red"))(length(bk)/2)),

cluster_rows =F,

cluster_cols =F,

scale="none",

fontsize = 15,

fontsize_row=15,

legend_breaks=seq(floor(min(outab)),ceiling(max(outab)),1),

breaks=bk,

gaps_row = c(8, 16),

main="Fig5b",

display_numbers = matrix(ifelse(ptab < 0.01, "*", ""), nrow(ptab)))

dev.off()

**Figure 5c**

setwd("")

exp=read.table("Fig5c_input1.txt", header=T,sep="\t",row.names=1,check.names=F)

TMB=read.table("Fig5c_input2.txt", header=T,sep="\t",row.names=1,check.names=F)

group=sapply(strsplit(row.names(exp),"\\-"),"[",4)

group=sapply(strsplit(group,""),"[",1)

group=gsub("2","1",group)

exp=exp[group==0,]

sameSample=intersect(row.names(TMB),row.names(exp))

TMB=TMB[sameSample,]

exp=exp[sameSample,]

outTab=data.frame()

fmsbTab=data.frame()

cancers <- levels(factor(exp[,"CancerType"]))

for(i in cancers){

exp1=exp[(exp[,"CancerType"]==i),]

TMB1=TMB[(TMB[,"CancerType"]==i),]

x=as.numeric(TMB1[,1])

y=as.numeric(exp1[,1])

corT=cor.test(x,y,method="spearman")

cor=corT$estimate

pValue=corT$p.value

sig=ifelse(pValue<0.001,"***",ifelse(pValue<0.01,"**",ifelse(pValue<0.05,"*"," ")))

outTab=rbind(outTab,cbind(CancerType=i,cor=cor,pValue=pValue,sig))

fmsbTab=rbind(fmsbTab,cbind(CancerType=i,cor=cor))

}

write.table(outTab,file="corStat.txt",sep="\t",row.names=F,quote=F)

write.table(t(fmsbTab),file="fmsbInput.txt",sep="\t",col.names=F,quote=F)

library(fmsb)

data=read.table("fmsbInput.txt",header=T,sep="\t",row.names=1,check.names=F)

maxValue=ceiling(max(abs(data))*10)/10

data=rbind(rep(maxValue,ncol(data)),rep(-maxValue,ncol(data)),data)

colors="red"

corStat=read.table("corStat.txt",header=T,sep="\t",row.names=1,check.names=F)

colnames(data)=paste0(colnames(data),corStat$sig)

data=t(data)

data=as.data.frame(data)

data=data[order(-data$cor),]

data=t(data)

data=as.data.frame(data)

par(mar=c(2,2,2,2))

pdf(file="Fig5c.pdf",height=8,width=8)

radarchart(data, axistype=1 ,

pcol=colors,

plwd=2 ,

plty=1,

cglcol="grey",

cglty=1,

caxislabels=seq(-maxValue,maxValue,maxValue/2),

cglwd=1.2,

axislabcol="blue",

vlcex=0.8,

calcex=0.8,

title="PDL-1"

)

dev.off()

**Figure 5d**

setwd(" ")

exp=read.table("Fig5d_input1.txt", header=T,sep="\t",row.names=1,check.names=F)

MSI=read.table("Fig5d_input2.txt", header=T,sep="\t",row.names=1,check.names=F)

group=sapply(strsplit(row.names(exp),"\\-"),"[",4)

group=sapply(strsplit(group,""),"[",1)

group=gsub("2","1",group)

exp=exp[group==0,]

sameSample=intersect(row.names(MSI),row.names(exp))

MSI=MSI[sameSample,]

exp=exp[sameSample,]

cancers <- levels(factor(exp[,"CancerType"]))

outTab=data.frame()

fmsbTab=data.frame()

for(i in cancers){

exp1=exp[(exp[,"CancerType"]==i),]

MSI1=MSI[(MSI[,"CancerType"]==i),]

x=as.numeric(MSI1[,1])

y=as.numeric(exp1[,1])

corT=cor.test(x,y,method="spearman",exact = F)

cor=corT$estimate

pValue=corT$p.value

sig=ifelse(pValue<0.001,"***",ifelse(pValue<0.01,"**",ifelse(pValue<0.05,"*"," ")))

outTab=rbind(outTab,cbind(CancerType=i,cor=cor,pValue=pValue,sig))

fmsbTab=rbind(fmsbTab,cbind(CancerType=i,cor=cor))

}

write.table(outTab,file="corStat.txt",sep="\t",row.names=F,quote=F)

write.table(t(fmsbTab),file="fmsbInput.txt",sep="\t",col.names=F,quote=F)

library(fmsb)

data=read.table("Fig5d_input3.txt",header=T,sep="\t",row.names=1,check.names=F)

maxValue=ceiling(max(abs(data))*10)/10

data=rbind(rep(maxValue,ncol(data)),rep(-maxValue,ncol(data)),data)

colors="red"

corStat=read.table("Fig5d_input4.txt",header=T,sep="\t",row.names=1,check.names=F)

colnames(data)=paste0(colnames(data),corStat$sig)

data=t(data)

data=as.data.frame(data)

data=data[order(-data$cor),]

data=t(data)

data=as.data.frame(data)

par(mar=c(2,2,2,2))

pdf(file="Fig5d.pdf",height=8,width=8)

radarchart(data, axistype=1 ,

pcol=colors,

plwd=2 ,

plty=1,

cglcol="grey",

cglty=1,

caxislabels=seq(-maxValue,maxValue,maxValue/2),

cglwd=1.2,

axislabcol="blue",

vlcex=0.8,

calcex=0.8,

title="MSI"

)

dev.off()

**Figure 5e**

setwd("")

exp=read.table("Fig5e_input1.txt", header=T,sep="\t",row.names=1,check.names=F)

#??ȡTMB?ļ?

TMB=read.table("Fig5e_input2.txt", header=T,sep="\t",row.names=1,check.names=F)

group=sapply(strsplit(row.names(exp),"\\-"),"[",4)

group=sapply(strsplit(group,""),"[",1)

group=gsub("2","1",group)

exp=exp[group==0,]

sameSample=intersect(row.names(TMB),row.names(exp))

TMB=TMB[sameSample,]

exp=exp[sameSample,]

cancers <- levels(factor(exp[,"CancerType"]))

outTab=data.frame()

fmsbTab=data.frame()

for(i in cancers){

exp1=exp[(exp[,"CancerType"]==i),]

TMB1=TMB[(TMB[,"CancerType"]==i),]

x=as.numeric(TMB1[,1])

y=as.numeric(exp1[,1])

corT=cor.test(x,y,method="spearman",exact=FALSE)

cor=corT$estimate

pValue=corT$p.value

sig=ifelse(pValue<0.001,"***",ifelse(pValue<0.01,"**",ifelse(pValue<0.05,"*"," ")))

outTab=rbind(outTab,cbind(CancerType=i,cor=cor,pValue=pValue,sig))

fmsbTab=rbind(fmsbTab,cbind(CancerType=i,cor=cor))

}

write.table(outTab,file="corStat.txt",sep="\t",row.names=F,quote=F)

write.table(t(fmsbTab),file="fmsbInput.txt",sep="\t",col.names=F,quote=F)

library(fmsb)

data=read.table("fmsbInput.txt",header=T,sep="\t",row.names=1,check.names=F)

maxValue=ceiling(max(abs(data))*10)/10

data=rbind(rep(maxValue,ncol(data)),rep(-maxValue,ncol(data)),data)

colors="red"

corStat=read.table("corStat.txt",header=T,sep="\t",row.names=1,check.names=F)

colnames(data)=paste0(colnames(data),corStat$sig)

data=t(data)

data=as.data.frame(data)

data=data[order(-data$cor),]

data=t(data)

data=as.data.frame(data)

par(mar=c(2,2,2,2))

pdf(file="Fig5e.pdf",height=8,width=8)

radarchart(data, axistype=1 ,

pcol=colors,

plwd=2 ,

plty=1,

cglcol="grey",

cglty=1,

caxislabels=seq(-maxValue,maxValue,maxValue/2),

cglwd=1.2,

axislabcol="blue",

vlcex=0.8,

calcex=0.8,

title="TMB"

)

dev.off()

**Figure 6a**

library(vioplot)

setwd("")

rt=read.table("Fig6a_input.txt",sep="\t",header=T,row.names=1,check.names=F)

rt=data.frame(rt)

group=levels(factor(rt$CancerType))

group=c("KIRP","ESCA","GBM","KIRC","THCA","LUAD","LUSC","PCPG","BLCA","KICH") #for hot cancers

group=c("COAD","LIHC","CHOL","READ","BRCA","SARC") #for cold cancers

group=c( "KIRP","ESCA","GBM","KIRC","THCA","LUAD","LUSC","PCPG","BLCA","KICH","COAD","LIHC","CHOL","READ","BRCA","SARC")

outpdf="Fig6a.pdf"

pdf(outpdf,height=8,width=20)

par(las=1,mar=c(10,6,6,6))

x=c(1:16)

y=c(1:16)

plot(x,y,

xlim=c(0,34),ylim=c(min(rt[,1]),max(rt[,1])+0.2),

main="",xlab="", ylab="relative mRNA expression",

pch=21,

col="white",

xaxt="n")

text(seq(1,36,2.22),-0.1,xpd = NA,labels=group,cex = 1,srt = 45,pos=2)

text(30,1.7,xpd = NA,labels="lasso(ssgesa) between nomral and tumor",cex = 1.5,pos=2)

for (j in group) {

rt1=rt[rt[,"CancerType"]==j,]

count=which(group==j)

normal=rt1[rt1[,"Type"]=="Normal",]

normal=as.data.frame(normal)

tumor= rt1[rt1[,"Type"]=="Tumor",]

tumor=as.data.frame(tumor)

normalData=as.numeric(normal[,1])

tumorData=as.numeric(tumor[,1])

vioplot(normalData,at=2.2*(count-1),lty=1,add = T,col = 'blue')

vioplot(tumorData,at=2.2*(count-1)+1,lty=1,add = T,col = 'red')

wilcoxTest=wilcox.test(normalData,tumorData,exact=FALSE)

p=round(wilcoxTest$p.value,3)

mx=max(c(normalData,tumorData))

lines(c(x=2.2*(count-1)+0.2,x=2.2*(count-1)+0.8),c(mx,mx))

text(x=2.2*(count-1)+0.5,y=mx+0.2,labels=ifelse(p<0.001,paste0("p<0.001"),paste0("p=",p)),cex = 1.0)

}

dev.off()

**Figure 6b-d**

library(dplyr)

library(pROC)

setwd("")

rt <- read.table("Fig6b-c_input.txt",header=T,sep="\t",check.names=F)

rt <- rt %>% select(ferroptosis,Type,CancerType)

cancertype <- levels(factor(rt$CancerType))

for (i in cancertype) {

data <- rt[rt$CancerType==i,]

roc1 <- roc(data$Type, data$ferroptosis,ci=TRUE)

levels(data$Type) <- c(`Normal`=0,`Tumor`=1)

# ci(roc1)

# ci(roc1, of="auc")

# ci.auc(roc1)

# roc2 <- roc(rt$GROUP, rt$`Inflammatory Caspases`)

# roc3 <- roc(rt$GROUP, rt$`Gasdermin Family`)

# roc4 <- roc(rt$GROUP, rt$`Pro-inflammatory Cytokines`)

auc(roc1)

ci(roc1,of="auc")

# auc(roc2)

# auc(roc3)

# auc(roc4)

outfile <- paste0("ROC_",i,".pdf")

pdf(file=outfile,width=6,height=6)

# par(oma=c(0.5,1,0,1),font.lab=1.5,font.axis=1.5)

plot.roc(roc1, col="red",print.auc=T,print.auc.pattern=NULL,legacy.axes=T)

# plot(roc1$specificities, roc1$sensitivities, type="l", xlim=c(0,1), ylim=c(0,1),col='red',

# xlab="specificity", ylab="sensitivity",

# main=paste("ROC curve (", "AUC = ",round(roc1$auc,3),")"),

# lwd = 2, cex.main=1.3, cex.lab=1.2, cex.axis=1.2, font=1.2)

# plot.roc(roc2, add=TRUE, col="blue")

# plot.roc(roc3, add=TRUE, col="green")

# plot.roc(roc4, add=TRUE, col="orange")

par(mar=c(0,0,0,2),xpd = T,cex.axis=1.6)

legend("topright",

legend="lasso(ssgesa)",

col=c("red","blue","green","orange"),

title=i,

lty=1,lwd=2,xpd=TRUE)

print(plot)

dev.off()

}

**Figure S1C**

library(vioplot)

setwd("")

files=grep(".txt",dir(),value = T)

for (i in files) {

rt=read.table(i,sep="\t",header=T,row.names=1,check.names=F)

rt=data.frame(rt)

genename=sapply(strsplit(i,".txt"),"[",1)

group=levels(factor(rt$CancerType))

ymax=max(rt[,1])

outfile=paste0(genename,".pdf")

pdf(outfile,height=8,width=20)

par(las=1,mar=c(10,6,3,3))

x=c(1:19)

y=c(1:19)

plot(x,y,

xlim=c(0,36),ylim=c(min(rt[,1]),max(rt[,1])+0.5),

main="",xlab="", ylab="relative mRNA expression levels",cex.lab = 2,

pch=21,

col="white",

xaxt="n")

text(seq(1,37,2.22),-ymax/10,xpd = NA,labels=group,cex = 1.7,srt = 45,pos=2)

text(19,ymax*1.15,xpd = NA,labels="GSE98384",cex = 2,pos=2)

legend(1,ymax*1, c('normal','tumor'), col = c("blue", "red"),text.col ='black', pch = c(15, 15 ), bg ='white',pt.cex=1.6,cex=1.6)

for (j in group) {

rt1=rt[rt[,"CancerType"]==j,]

count=which(group==j)

normal=rt1[rt1[,"Type"]=="normal",]

normal=as.data.frame(normal)

tumor= rt1[rt1[,"Type"]=="tumor",]

tumor=as.data.frame(tumor)

normalData=as.numeric(normal[,1])

tumorData=as.numeric(tumor[,1])

vioplot(normalData,at=2.2*(count-1),lty=1,add = T,col = 'blue')

vioplot(tumorData,at=2.2*(count-1)+1,lty=1,add = T,col = 'red')

wilcoxTest=wilcox.test(normalData,tumorData,exact=FALSE)

p=round(wilcoxTest$p.value,3)

mx=max(c(normalData,tumorData))

lines(c(x=2.2*(count-1)+0.2,x=2.2*(count-1)+0.8),c(mx,mx))

text(x=2.2*(count-1)+0.5,y=mx+0.5,labels=ifelse(p<0.001,paste0("****"),ifelse(p<0.01,paste0("***"),paste0("NS"))),cex = 1.6)

}

dev.off()

}

**Figure S2AB**

#install.packages("glmnet")

#install.packages("survival")

library("glmnet")

library("survival")

setwd("")

rt=read.table("FigS2_input.txt",header=T,sep="\t",row.names=1)

rt$futime=rt$futime/365

x=as.matrix(rt[,c(3:ncol(rt))])

y=data.matrix(Surv(rt$futime,rt$fustat))

fit <- glmnet(x, y, family = "cox", maxit = 9000)

pdf("FigS2b.pdf")

plot(fit, xvar = "lambda", label = TRUE)

dev.off()

cvfit <- cv.glmnet(x, y, family="cox", maxit = 9000)

pdf("FigS2a.pdf")

plot(cvfit)

abline(v=log(c(cvfit$lambda.min,cvfit$lambda.1se)),lty="dashed")

dev.off()

coef <- coef(fit, s = cvfit$lambda.min)

index <- which(coef != 0)

actCoef <- coef[index]

lassoGene=row.names(coef)[index]

geneCoef=cbind(Gene=lassoGene,Coef=actCoef)

write.table(geneCoef,file="geneCoef.txt",sep="\t",quote=F,row.names=F)

riskScore=predict(cvfit, newx = x, s = "lambda.min",type="response")

outCol=c("futime","fustat",lassoGene)

risk=as.vector(ifelse(riskScore>median(riskScore),"high","low"))

outTab=cbind(rt[,outCol],riskScore=as.vector(riskScore),risk)

write.table(cbind(id=rownames(outTab),outTab),

file="lassoRisk.txt",

sep="\t",

quote=F,

row.names=F)

#install.packages("survivalROC")

**Figure S2C**

#install.packages('survival')

#install.packages('forestplot')

setwd("C:\\Users\\Administrator\\Desktop\\test")

library(survival)

library(forestplot)

options(forestplot_new_page = FALSE)

clrs <- fpColors(box="green",line="darkblue", summary="royalblue")

rt=read.table("cox.txt",header=T,sep="\t",check.names=F,row.names=1)

outTab=data.frame()

for(i in colnames(rt[,3:ncol(rt)])){

cox <- coxph(Surv(futime, fustat) ~ rt[,i], data = rt)

coxSummary = summary(cox)

coxP=coxSummary$coefficients[,"Pr(>|z|)"]

outTab=rbind(outTab,

cbind(id=i,

HR=coxSummary$conf.int[,"exp(coef)"],

HR.95L=coxSummary$conf.int[,"lower .95"],

HR.95H=coxSummary$conf.int[,"upper .95"],

pvalue=coxSummary$coefficients[,"Pr(>|z|)"])

)

}

write.table(outTab,file="uniCox.xls",sep="\t",row.names=F,quote=F)

rt=read.table("uniCox.xls",header=T,sep="\t",row.names=1,check.names=F)

data=as.matrix(rt)

HR=data[,1:3]

hr=sprintf("%.3f",HR[,"HR"])

hrLow=sprintf("%.3f",HR[,"HR.95L"])

hrHigh=sprintf("%.3f",HR[,"HR.95H"])

pVal=data[,"pvalue"]

pVal=ifelse(pVal<0.001, "<0.001", sprintf("%.3f", pVal))

tabletext <-

list(c(NA, rownames(HR)),

append("pvalue", pVal),

append("Hazard ratio",paste0(hr,"(",hrLow,"-",hrHigh,")")) )

pdf(file="forest.pdf",

width = 8,

height = 8,

)

forestplot(tabletext,

rbind(rep(NA, 3), HR),

col=clrs,

graphwidth=unit(50, "mm"),

xlog=T,

lwd.ci=2,

boxsize=0.3,

xlab="Hazard ratio",

title="SKCM"

)

dev.off()

**Figure S2d-k**

#install.packages("survival")

setwd("")

library(survival)

pFilter=1

rt=read.table("FigS2d-k_input.txt",header=T,sep="\t",check.names=F)

rt$futime=rt$futime/365

outTab=data.frame()

for(gene in colnames(rt[,4:ncol(rt)])){

if(sd(rt[,gene])==0){

next}

a=rt[,gene]<=median(rt[,gene])

diff=survdiff(Surv(futime, fustat) ~a,data = rt)

pValue=1-pchisq(diff$chisq,df=1)

outTab=rbind(outTab,cbind(gene=gene,pvalue=pValue))

fit <- survfit(Surv(futime, fustat) ~ a, data = rt)

summary(fit)

if(pValue<pFilter){

if(pValue<0.001){

pValue=signif(pValue,4)

pValue=format(pValue, scientific = TRUE)

}else{

pValue=round(pValue,3)

}

pdf(file=paste(gene,".survival.pdf",sep=""),

width = 5.5,

height =5,

)

plot(fit,

lwd=2,

col=c("red","blue"),

xlab="Time (year)",

mark.time=T,

ylab="Survival rate",

ylim=c(0,1.09),

main=paste("TCGA (p=", pValue ,")",sep=""))

legend("topright",

c(paste(gene," high expression",sep=""),

paste(gene," low expression",sep="") ),

lwd=2,

col=c("red","blue"))

dev.off()

}

}

write.table(outTab,file="survival.xls",sep="\t",row.names=F,quote=F)
